# The Surprise Question and clinician-predicted prognosis: systematic review and meta-analysis

**DOI:** 10.1136/spcare-2024-004879

**Published:** 2024-06-26

**Authors:** Ankit Gupta, Ruth Burgess, Michael Drozd, John Gierula, Klaus Witte, Sam Straw

**Affiliations:** 1 Leeds Institute of Medical Education, University of Leeds, Leeds, UK; 2 Leeds Teaching Hospitals NHS Trust, Leeds, UK; 3 Leeds Institute of Cardiovascular and Metabolic Medicine, University of Leeds, Leeds, UK

**Keywords:** Palliative Care, Prognosis

## Abstract

**ABSTRACT:**

**Background:**

The Surprise Question, ‘Would you be surprised if this person died within the next year?’ is a simple tool that can be used by clinicians to identify people within the last year of life. This review aimed to determine the accuracy of this assessment, across different healthcare settings, specialties, follow-up periods and respondents.

**Methods:**

Searches were conducted of Medline, Embase, AMED, PubMed and the Cochrane Central Register of Controlled Trials, from inception until 01 January 2024. Studies were included if they reported original data on the ability of the Surprise Question to predict survival. For each study (including subgroups), sensitivity, specificity, positive and negative predictive values and accuracy were determined.

**Results:**

Our dataset comprised 56 distinct cohorts, including 68 829 patients. In a pooled analysis, the sensitivity of the Surprise Question was 0.69 ((0.64 to 0.74) I^2^=97.2%), specificity 0.69 ((0.63 to 0.74) I^2^=99.7%), positive predictive value 0.40 ((0.35 to 0.45) I^2^=99.4%), negative predictive value 0.89 ((0.87 to 0.91) I^2^=99.7%) and accuracy 0.71 ((0.68 to 0.75) I^2^=99.3%). The prompt performed best in populations with high event rates, shorter timeframes and when posed to more experienced respondents.

**Conclusions:**

The Surprise Question demonstrated modest accuracy with considerable heterogeneity across the population to which it was applied and to whom it was posed. Prospective studies should test whether the prompt can facilitate timely access to palliative care services, as originally envisioned.

**PROSPERO registration number:**

CRD32022298236.

**WHAT IS ALREADY KNOWN ON THIS TOPIC**

- The Surprise Question is a simple tool that could help identify people within the last year of life.
- Current evidence suggests that the Surprise Question has reasonable accuracy for identifying patients at higher risk of mortality, potentially aiding clinicians to initiate timely discussions about palliative and end-of-life care.

**WHAT THIS STUDY ADDS**

- The Surprise Question has modest accuracy for identifying those nearing the end of life with some inconsistency across settings, specialities and follow-up times.
- The prompt performs best when used in populations with high event rates, when posed over shorter timeframes, and when used in inpatient settings.

**HOW THIS STUDY MIGHT AFFECT RESEARCH, PRACTICE OR POLICY**

- Our meta-analysis helps further refine the role of the Surprise Question as a prognostic tool in acute and chronic illnesses.
- Future research should address whether integrating the Surprise Question into routine clinical care improves access to palliative care services, facilitates advance care planning and is acceptable to the healthcare team.

## Introduction

 The Surprise Question, ‘Would you be surprised if this person were to die within the next year?’ is a simple prompt, originally developed to help healthcare professionals identify patients who are nearing the end of life who might require additional support and access to palliative care services.^1^ Anticipated prognosis is a major driver of these decisions, such that the ability of the Surprise Question to identify those within the last year of life has been assessed across a diverse range of healthcare settings. Although clinician-predicted prognosis is simple and convenient, it may lack accuracy due to a tendency to overestimate survival.^2^ The Surprise Question aims to address this tendency by posing a reflective question as to whether death is possible, rather than likely.^3^

The Surprise Question is a core component of the Gold Standards Framework tool in the United Kingdom which is recommended for use across primary and secondary healthcare settings to identify those nearing the end of life.^4^ The use of the Surprise Question to identify those in the last year of life is also endorsed in position statements from both the American Heart Association^5^ and Japanese Cardiology Society/Heart Failure Society.^6^ Despite its widespread use, the prognostic accuracy of the Surprise Question is uncertain and may depend on the setting in which it is applied, the disease studied, timeframe chosen, event (death) rate in the population and to whom the question is posed.^7^

Previous meta-analyses^8 9^ have not included studies using shorter timeframes, or have not considered the accuracy of the Surprise Question when utilised in different healthcare settings.^10^ We first aimed to provide an updated systematic review and meta-analysis of the accuracy of the Surprise Question. Second, we aimed to assess the accuracy of the Surprise Question across populations with different event rates, healthcare settings, specialties, timeframes and when posed to different healthcare professionals.

## Methods

In accordance with the Preferred Reporting Items for Systematic Review and Meta-analysis study guidelines, our study aimed to assess the accuracy of the Surprise Question.^11^

### Search strategy

The study protocol was registered with PROSPERO (online supplemental file 1). We searched for articles indexed in Medline, Embase, Allied and Complimentary Medicine Database (AMED), PubMed, Cochrane Database of Systematic Reviews, and the Cochrane Central Register of Controlled Trials from inception until 01 January 2024, including articles being processed at that time. The full search strategy is available in table 1. Briefly, we searched the literature using variations of the search terms “Surprise Question” and “mortality” or “Gold standards Framework” and “mortality”. Additionally, the references of all included articles and review articles were assessed manually to identify any additional relevant publications. We limited our search to studies on human subjects, including both adult and paediatric populations, and to articles published in English, or for which an English translation was available. No other filters were applied.

**Table 1:** Search strategy

Database	Search terms	Studies identified
OVID – MEDLINE, Embase, AMED, HMIC, Emcare	(((Surprise OR surprize OR surprising OR surprised) AND (question or questions) AND (dying OR death OR mortality OR survival OR die OR outcome OR outcomes OR palliative OR end of life))**OR**((GSF OR gold standards framework) AND (dying OR death OR mortality OR survival OR die OR outcome OR outcomes OR palliative OR end of life)))	2295
PubMed	(((Surprise OR surprize OR surprising OR surprised) AND (question or questions) AND (dying OR death OR mortality OR survival OR die OR outcome OR outcomes OR palliative OR end of life))**OR**((GSF OR gold standards framework) AND (dying OR death OR mortality OR survival OR die OR outcome OR outcomes OR palliative OR end of life)))	1535
CDSR	(((Surprise OR surprize OR surprising OR surprised) AND (question or questions) AND (dying OR death OR mortality OR survival OR die OR outcome OR outcomes OR palliative OR end of life))**OR**((GSF OR gold standards framework) AND (dying OR death OR mortality OR survival OR die OR outcome OR outcomes OR palliative OR end of life)))	21
CCRCT	(((Surprise OR surprize OR surprising OR surprised) AND (question or questions) AND (dying OR death OR mortality OR survival OR die OR outcome OR outcomes OR palliative OR end of life))**OR**((GSF OR gold standards framework) AND (dying OR death OR mortality OR survival OR die OR outcome OR outcomes OR palliative OR end of life)))	207

### Study selection

AG performed the search and SS adjudicated the search strategy before and during the time it was applied to the respective databases. Studies identified from database searches were screened independently by AG and SS. The first selection criterion was that the title or abstract included either ‘Surprise Question’ or ‘Gold Standards Framework’ with any study not meeting this criterion excluded. We placed no restrictions on study design, although at full review we required studies to report mortality data divided by whether patients received a ‘surprised’ or a ‘not surprised’ response from a healthcare professional and, therefore, all studies were prospective and observational in nature. We excluded studies where it was not possible to determine the sensitivity, specificity, positive and negative predictive values (NPV) and accuracy of the Surprise Question for the population studied. Where these data were unavailable, but the article appeared potentially relevant, applications for raw data were made to corresponding authors. No restrictions were placed on the setting, disease studied, timeframe evaluated or healthcare professional providing the response. Any discrepancies were resolved by meeting between AG and SS. The option for unresolved discrepancies to be adjudicated by a third reviewer (KW) was never required.

### Quality assessment of studies

Each study was assessed independently by AG and SS who met and discussed the study designs. As studies were observational in nature, each rater independently completed the Newcastle-Ottawa Scale. Any discrepancies could be adjudicated by a third reviewer (KW), although none was. The Newcastle-Ottawa Scale rates observational studies based on three domains: selection, comparability between the exposed and unexposed groups and exposure/outcome assessment. The scale assigns a maximum of four stars for selection, two for comparability and three for exposure/outcome. In line with the Agency for Healthcare Research and Quality standards, the quality of the studies was categorised into either good, fair or poor. Good-quality articles were those which received three or four stars in the selection domain, and one or two stars in the comparability domain and two or three stars in the exposure/outcome domain. Fair-quality articles received two stars in the selection domain, and one or two stars in the comparability domain and two or three stars in the exposure/outcome domain. Finally, those which were of poor quality received either 0 or one star in the selection domain, or 0 stars in the comparability, or 0 or one stars in the exposure/outcome domain.

### Data extraction

Data from included studies were extracted independently by AG and SS, who recorded study design, setting (primary care, outpatient, emergency department or inpatient), medical or surgical specialty, number of patients, timeframe assessed and type of healthcare professionals providing responses. Where data were reported from separate participants, responses were pooled into an overall estimate, with responses from other healthcare professionals then assessed separately in subgroup analyses. Where data were reported from separate time points from the same cohort, these were analysed separately.

Two-by-two tables were compiled for each study and relevant subgroups to determine the predictive value of the Surprise Question. The sensitivity was the proportion of patients who received a ‘not surprised’ response and subsequently died, whereas the specificity was the proportion of patients who received a ‘surprised’ response and subsequently died. The positive predictive value (PPV) was the proportion of patients who received a ‘not surprised’ response and subsequently died, and the NPV was the proportion of patients who received a ‘surprised’ response and subsequently survived. Accuracy was the proportion of patients correctly predicted by the Surprise Question. These are presented alongside 95% CIs for the overall comparisons of each study and subgroup analyses, with heterogeneity estimated by the I^2^ statistic. Event (death) rates were calculated for each study by dividing the total number of deaths by the total cohort size, expressed as a percentage.

### Data analysis

We synthesised estimates of the accuracy of the Surprise Question using a random effects meta-analysis model using the meta-analysis function in STATA V.16 (StataCorp LLC, College Station, Texas). We used the restricted maximum-likelihood model, which was used for calculating τ^2^. Overall comparisons were calculated, and then where appropriate, studies were divided by event rate, setting, specialty, timeframe of follow-up and healthcare professional. Where the Surprise Question was reported separately from different healthcare professional groups, we pooled responses to calculate an overall estimate (if this was not provided in the manuscript) with individual group responses recorded separately. Where studies reported responses from timeframes other than 1 year, these estimates were not included in the overall comparisons and were reported separately.

## Results

**Figure 1:**
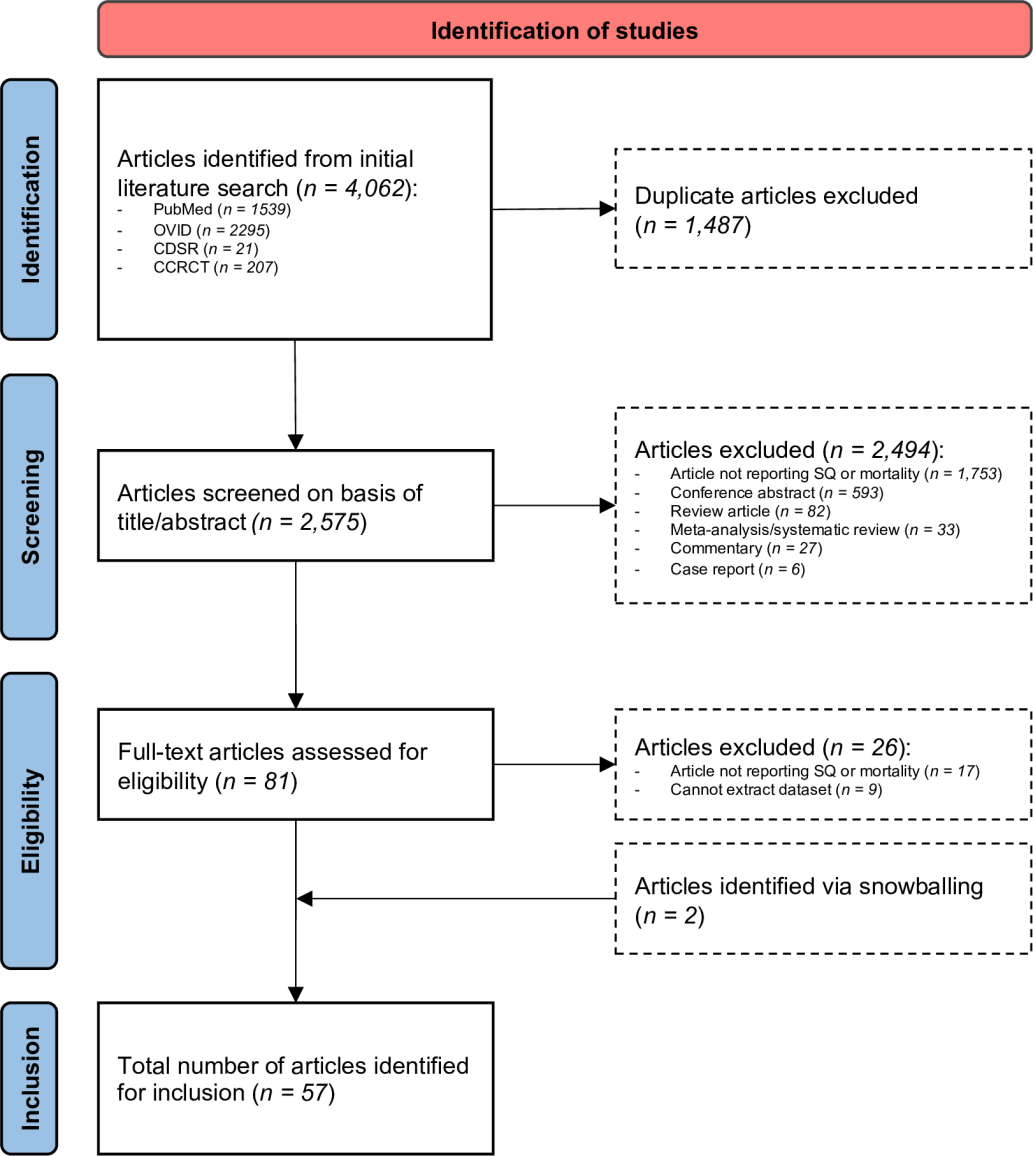
PRISMA flow diagram of article screening process. PRISMA, Preferred Reporting Items for Systematic Review and Meta-analysis.

The search of four electronic databases identified 4062 records, with 2575 articles remaining after the removal of duplicates. Of these, 2494 were excluded after the screening of the titles and abstracts, usually because they were not relevant or did not report original data (figure 1). Of the 81 retrieved articles, 26 were excluded after full-text review because data were not available to calculate the accuracy of the Surprise Question even after request to the corresponding author. Two articles were identified from the references of included studies. A total of 57 studies met the full inclusion criteria, however two reported data from the same population but using different timeframes. Our final dataset, therefore, consisted of 56 distinct cohorts, including a total of 68 829 unique patients.

### Study characteristics

The characteristics of the individual studies are displayed in table 2, all of which were prospective, observational cohort studies. Most studies reported data from adult patients (although the age and sex were often not reported). One study was conducted in a paediatric population. The majority of studies reported data from Europe or the USA, but the dataset included studies from all global regions. Forty-two studies chose a timeframe of 1 year to assess the prognostic accuracy of the Surprise Question, with other studies reporting the accuracy between 1 day and 3.3 years. Twenty-three (41.1%) studies were conducted in outpatient settings, 16 (28.6%) in hospitalised patients, 7 (12.5%) in primary care, 5 (8.9%) in the emergency department and 5 (8.9%) in community settings.

**Table 2:** Characteristics of studies included in systematic review

First author	Year	Country	Time	Setting	Specialty	Respondent(s)	Patients	Patient age(years)	Male patients (%)
Mahes A.	2023	USA	1 year	Hospital outpatient	Unselected	Physicians	301	74.7±8.2	67.1
Lin C.A.	2023	Brazil	1 year	Hospital outpatient	General medicine	Physicians	840	60.9±14.9	31.9
Um Y.W.	2023	South Korea	30-days	Emergency department	Unselected	Physicians	300	70.5 (59.3–81.8)	54
DogbeyD.M.	2022	South Africa	6 months	Inpatient	Oncology	Physicians	43	58 (45–68)	35
Kim S.H.	2022	South Korea	7 days21-days42-days	Inpatient	Oncology	Physicians	130	66.0±12.2	50.8
Maes H.	2022	Belgium	1 year	Hospital inpatient	UnselectedCardiology	Physicians	381	Not reported	81.4
Gaffney L.	2022	Ireland	1 year	Emergency department	Unselected	Physicians	191	79 (74–83)	45
Ikari T.	2021	Japan, Korea, Taiwan	1 day	Inpatient	Oncology	Physicians	1411	72.6±12.2	50.7
Ikari T.	2021	Japan, Korea, Taiwan	3 days	Inpatient	Oncology	Physicians	1411	72.6±12.2	50.7
Moor C.C.	2021	Netherlands	1 year	Outpatient	Respiratory	PhysiciansSpecialist nurses	140	74.0±6.5	87.1
Gonzalez- Jaramillo V.	2021	Colombia	1 year	Outpatient	Cardiology	Physicians	174	70 (58–77)	55.2
Tripp D.	2021	USA	30 days1-year	Inpatient	Respiratory	PhysiciansAdvanced practice providers	428	Not reported	49.1
ErmersD.J.M.	2021	Netherlands	1 year	Hospital outpatient	Oncology	PhysiciansTrainee physicians	379	59.4±15	55.7
Yarnell C.	2021	Canada	1 year	Hospital inpatient	General medicine	PhysiciansTrainee physicians	417	75 (60–85)	52.0
Ros M.M.	2021	Netherlands	2 days10-days1-year	Intensive care unit	Unselected	Physicians	3140	63.5±16.6	57.1
Flierman I.	2020	Netherlands	1 year	Inpatient	Unselected	Nurses	234	81.2±6.6	48.4
Van WijmenM.P.S.	2020	Netherlands	1 year	Primary care	Unselected	Physicians	57	Not reported	28.4
Ramer S.J.	2020	USA	2 years	Hospital outpatient	Nephrology	PhysiciansAdvanced practitioners	377	72 (66–78)	49
Lai C.-F.	2020	Taiwan	1 year	Hospital outpatient	Nephrology	Nurses	401	56.2±14	49.9
Rauh L.A.	2020	USA	1 year	Hospital outpatient	Oncology	PhysicianNurseAdvanced practice providers	358	Not reported	Not reported
Yen Y.-F.	2020	Taiwan	1 year	Hospital inpatient	Unselected	Nurses	21 098	62.8±19.0	53.2
Ouchi K.	2019	USA	30-days	Emergency department	Unselected	Physicians	10 737	75.9±8.8	48.5
Verhoef M.J.	2019	Netherlands	1 year	Emergency department	Oncology	Physicians	245	62 (45–79)	48
AaronsonE.L.	2019	USA	1 year	Emergency department	Cardiology	Physicians	193	74.5±12.6	
SchmidtR.J.	2019	USA	1 year	Hospital outpatient	Nephrology	PhysiciansTrainee physiciansNurse practitioners	749	69.3±14.6	50.9
Lakin J.R.	2019	USA	2 years	Primary care	Primary care	PhysiciansNurses	2611	Not reported	40.9
VeldhovenC.M.M.	2019	Netherlands	1 year	Primary care	Unselected	Physicians	292	84±5.5	40.1
Haydar S.A.	2019	USA	30-days	Emergency department	Unselected	Physicians	6122	66 (51–79)	51.7
Straw S.	2019	UK	1 year	Hospital inpatient	Cardiology	PhysiciansTrainee-physiciansNursesNurse practitioners	129	71±14	64
Rice J.	2018	Canada	3 months	Nursing home	Unselected	Physicians	301	85.9±9.0	67.8
			6 months			NursesSupport workers			
Burke K.	2018	UK	90 days1-year	Hospice	Paediatric	Physicians Nurses Administrators	327	7.6±5.3	56.6
Ouchi K.	2018	USA	1 year	Emergency department	Unselected	Physicians	207	75±7.5	51.2
Liyanage T.	2018	Australia	1 year	Nursing home	Unselected	Nurses	187	82.4±9.1	44.4
MitchellG.K.	2018	Australia	1 year	Primary care	Unselected	Physicians	4365	Not reported	Not reported
Ebke M.	2018	Germany	1 year	Hospital outpatient	Neurorehabilitation	Physicians	236	63±14	57.7
Mudge A.M.	2018	Australia	1 year	Hospital inpatient	Unselected	PhysiciansNurses	100	60.2±18.9	53.8
GuliniJ.E.H.M.B.	2018	Brazil	1 day	Intensive care unit	Unselected	Physicians	170	57±15.6	51.2
Salat H.	2017	USA	1.9 years	Hospital outpatient	Nephrology	Physician	488	71 (65–77)	49
Malhotra R.	2017	USA	6 months1-year	Hospital outpatient	Nephrology	Physician	215	Not reported	55
Hadique S.	2017	USA	6 months	Intensive care unit	Unselected	Physician	1043	Not reported	53.7
Gomez- Batiste X.	2017	Spain	1 year2-years	Primary care Hospital outpatient Intermediate care centre Nursing home	Unselected	Physician/nurse	1059	Not reported	Not reported
Javier A.D.	2017	USA	1 year	Hospital outpatient	Nephrology	PhysiciansTrainee physiciansNurse practitioners	388	71 (65–77)	49.7
Amro O.W.	2016	USA	1 year	Hospital outpatient	Nephrology	Physicians	201	66	52.2
Lakin J.R.	2016	USA	1 year	Primary care	Unselected	Physicians	1737	65	39.7
Carmen J.	2016	Spain	1 year	Hospital outpatient	Nephrology	Physicians	49	Not reported	Not reported
Hamano J.	2015	Japan	7 days30-days	Hospital and community palliative care	Oncology	Physicians	2361	69.1±12.8	57.5
Feyi K.	2015	UK	1 year	Hospital outpatient	Nephrology	PhysiciansNurses	178	72	63.2
Moroni M.	2014	Italy	1 year	Primary care	Primary care/oncology	Physicians	231	70.2 (SE 0.9)	50.6
O'CallaghanA.	2014	New Zealand	6 months1-year	Hospital inpatient	Acute medicine	PhysiciansNurse practitioners	501	Not reported	Not reported
Da SilvaGane M.	2013	UK	1 year	Hospital outpatient	Nephrology	PhysicianNurses	3896	Not reported	Not reported
Pang W.-F.	2013	China	1 year	Hospital outpatient	Nephrology	Physicians	367	60.2±12.3	55.9
Haga K.	2012	UK	1 year	Hospital outpatient	Cardiology	Nurses	138	77±10	66
Fenning S.	2012	UK	1 year	Hospital outpatient	Cardiology	Physicians	172	66±14	61
Moss A.H.	2010	USA	1 year	Hospital outpatient	Oncology	Physicians	826	Not reported	Not reported
Cohen L.M.	2010	USA	6 months	Hospital outpatient	Nephrology	Physicians	450	Not reported	56.7
Moss A.H.	2008	USA	1 year	Hospital outpatient	Nephrology	Physicians	147	66.4±14.8	55.1
Barnes S.	2008	UK	1 year	Primary care	Cardiology	Physicians	231	77	54.1

Age is displayed as mean±SD deviation or median (IQR) where available.

In the pooled comparison across all 56 studies, the prognostic accuracy of the Surprise Question was modest, with high heterogeneity between studies. The accuracy of individual studies is shown in table 3. The sensitivity of a ‘not surprised’ response was 0.69 ((0.64 to 0.74) I^2^=97.2%), the specificity was 0.69 ((0.63 to 0.74) I^2^=99.7%), the PPV was 0.40 ((0.35 to 0.45) I^2^=99.4%), the NPV was 0.89 ((0.87 to 0.91) I^2^=99.7%), and the accuracy was 0.71 ((0.68 to 0.75) I^2^=99.3%).

**Table 3:** Accuracy of individual studies

First author	Sample size	Surprise question results				Diagnostic test results				
		Surprise Question responses	Total	Dead	Alive	Sensitivity (95% CI)	Specificity (95% CI)	Positive predictive value (95% CI)	Negative predictive value (95% CI)	Accuracy (95% CI)
Rice J.	301	Not surprised	191	62	134	0.646 (0.541–0.741)	0.544 (0.485–0.602)	0.316 (0.276–0.360)	0.825 (0.779–0.863)	0.569 (0.518–0.619)
		Surprised	194	34	160					
Ikari T.	1411	Not surprised	847	232	615	0.820 (0.770–0.863)	0.455 (0.425–0.4840	0.274 (0.259–0.289)	0.910 (0.886–0.929)	0.528 (0.502–0.554)
		Surprised	564	51	513					
Ikari T.	1411	Not surprised	1179	636	543	0.944 (0.923–0.960)	0.263 (0.232–0.297)	0.539 (0.528–0.551)	0.836 (0.786–0.877)	0.588 (0.562–0.614)
		Surprised	232	38	194					
Dogbey D.M.	43	Not surprised	21	18	3	0.667 (0.460–0.835)	0.813 (0.544–0.960)	0.857 (0.676–0.945)	0.591 (0.446–0.721)	0.721 (0.563–0.847)
		Surprised	22	9	13					
Moor C.C.	140	Not surprised	39	19	20	0.679 (0.477–0.841)	0.821 (0.738–0.887)	0.487 (0.372–0.604)	0.911 (0.856–0.946)	0.793 (0.716–0.857)
		Surprised	101	9	92					
Gonzalez- Jaramillo V.	174	Not surprised	83	17	66	0.850 (0.621–0.968)	0.571 (0.489–0.651)	0.205 (0.166–0.250)	0.967 (0.911–0.988)	0.603 (0.527–0.677)
		Surprised	91	3	88					
Tripp D.	381 (30 days)	Not surprised	19	2	17	0.125 (0.016–0.384)	0.953 (0.927–0.973)	0.105 (0.029–0.318)	0.961 (0.954–0.968)	0.919 (0.887–0.944)
		Surprised	362	14	348					
	365 (365 days)	Not surprised	108	38	70	0.469 (0.357–0.583)	0.754 (0.699–0.803)	0.352 (0.285–0.425)	0.833 (0.801–0.861)	0.690 (0.640–0.738)
		Surprised	257	43	214					
Flierman I.	234	Not surprised	135	59	76	0.808 (0.699–0.891)	0.528 (0.448–0.607)	0.437 (0.389–0.486)	0.859 (0.788–0.909)	0.615 (0.550–0.678)
		Surprised	99	14	85					
Kim S.H.	130 (7 days)	Not surprised	20	7	13	0.467 (0.213–0.734)	0.887 (0.815–0.938)	0.350 (0.204–0.531)	0.927 (0.888–0.954)	0.839 (0.764–0.897)
		Surprised	110	8	102					
	130 (21 days)	Not surprised	54	37	17	0.529 (0.406–0.649)	0.717 (0.586–0.826)	0.685 (0.579–0.775)	0.566 (0.493–0.636)	0.615 (0.526–0.699)
		Surprised	76	33	43					
	130 (42 days)	Not surprised	100	87	13	0.821 (0.734–0.889)	0.458 (0.256–0.672)	0.870 (0.821–0.907)	0.367 (0.242–0.512)	0.754 (0.671–0.825)
		Surprised	30	19	11					
Ouchi K.	10 737	Not surprised	3324	685	2639	0.433 (0.409–0.458)	0.820 (0.813–0.826)	0.206 (0.196–0.217)	0.931 (0.928–0.933)	0.782 (0.776–0.788)
		Surprised	12 899	896	12 003					
ErmersD.J.M.	379 (SQ1)	Not surprised	188	103	85	0.873 (0.799–0.927)	0.674 (0.614–0.731)	0.548 (0.501–0.594)	0.922 (0.879–0.950)	0.736 (0.689–0.780)
		Surprised	191	15	176					
	188 (SQ2)	Not surprised	105	42	63	0.408 (0.312–0.509)	0.259 (0.170–0.365)	0.400 (0.339–0.465)	0.265 (0.196–0.348)	0.340 (0.273–0.413)
		Surprised	83	61	22					
Yarnell C.	417 (AP – 365 days)	Not surprised	298	143	155	0.894 (0.835–0.937)	0.397 (0.337–0.460)	0.480 (0.452–0.508)	0.857 (0.789–0.906)	0.588 (0.539–0.635)
		Surprised	119	17	102					
		Not surprised	250	132	118	0.825	0.541	0.528	0.832	0.650
	417 (SMR – 365 days)	Surprised	167	28	139	(0.757–0.881)	(0.478–0.603)	(0.490–0.565)	(0.777–0.876)	(0.602–0.696)
	288 (AP - admission)	Not surprised	53	21	32	0.636 (0.451–0.796)	0.875 (0.828–0.913)	0.396 (0.303–0.498)	0.949 (0.922–0.967)	0.847 (0.800–0.887)
		Surprised	235	12	223					
	288 (SMR –admission)	Not surprised	47	16	31	0.485 (0.308–0.665)	0.878 (0.832–0.916)	0.340 (0.242–0.455)	0.930 (0.904–0.949)	0.833 (0.785–0.875)
		Surprised	241	17	224					
Van WijmenM.P.S.	57	Not surprised	19	18	1	0.692 (0.482–0.857)	0.968 (0.833–0.999)	0.947 (0.720–0.992)	0.790 (0.677–0.870)	0.842 (0.721–0.925)
		Surprised	38	8	30					
Ramer S.J.	377	Not surprised	124	45	79	0.672 (0.546–0.782)	0.745 (0.693–0.793)	0.363 (0.307–0.423)	0.913 (0.881–0.937)	0.732 (0.684–0.776)
		Surprised	253	22	231					
Lai C.-F.	401	Not surprised	34	18	16	0.529 (0.351–0.702)	0.956 (0.930–0.975)	0.529 (0.388–0.667)	0.956 (0.939–0.969)	0.920 (0.889–0.945)
		Surprised	367	16	351					
Rauh L.A.	231 (MD – UPMC)	Not surprised	90	36	54	0.706 (0.562–0.825)	0.700 (0.627–0.766)	0.400 (0.334–0.470)	0.894 (0.845–0.929)	0.701 (0.638–0.760)
		Surprised	141	15	126					
	168 (RN – UPMC)	Not surprised	61	31	30	0.689 (0.534–0.818)	0.756 (0.671–0.829)	0.508 (0.417–0.599)	0.869 (0.810–0.912)	0.738 (0.665–0.803)
		Surprised	107	14	93					
	199 (APP – UPMC)	Not surprised	81	35	46	0.796 (0.647–0.902)	0.703 (0.625–0.774)	0.432 (0.364–0.503)	0.924 (0.870–0.956)	0.724 (0.656–0.785)
		Surprised	118	9	109					
	78 (MD – UVA)	Not surprised	39	19	20	0.864 (0.651–0.971)	0.643 (0.504–0.766)	0.487 (0.392–0.584)	0.923 (0.805–0.972)	0.705 (0.591–0.803)
		Surprised	39	3	36					
	130 (RN – UVA)	Not surprised	82	29	53	0.725 (0.561–0.854)	0.411 (0.308–0.520)	0.354 (0.297–0.414)	0.771 (0.658–0.855)	0.508 (0.419–0.596)
		Surprised	48	11	37					
	22 (APP – UVA)	Not surprised	17	8	9	1.000 (0.631–1.000)	0.357 (0.128–0.649)	0.471 (0.376–0.568)	1.000	0.591 (0.364–0.793)
		Surprised	5	0	5					
	309 (MD –combined)	Not surprised	129	55	74	0.753 (0.639–0.847)	0.686 (0.623–0.745)	0.426 (0.371–0.483)	0.900 (0.857–0.931)	0.702 (0.648–0.753)
		Surprised	180	18	162					
	298 (RN –combined)	Not surprised	143	60	83	0.706 (0.597–0.800)	0.610 (0.541–0.676)	0.420 (0.368–0.473)	0.839 (0.786–0.880)	0.638 (0.580–0.692)
		Surprised	155	25	130					
	221 (APP –combined)	Not surprised	98	43	55	0.827 (0.697–0.918)	0.675 (0.598–0.745)	0.439 (0.378–0.501)	0.927 (0.874–0.959)	0.710 (0.646–0.769)
		Surprised	123	9	114					
Verhoef M.- J.	245	Not surprised	203	172	31	0.891 (0.839–0.931)	0.404 (0.270–0.549)	0.847 (0.815–0.875)	0.500 (0.373–0.628)	0.788 (0.731–0.837)
		Surprised	42	21	21					
Maes H.	190 (AcuteGeriatric Unit)	Not surprised	66	31	35	0.674 (0.520–0.805)	0.757 (0.679–0.825)	0.470 (0.384–0.557)	0.879 (0.826–0.918)	0.737 (0.668–0.798)
		Surprised	124	15	109					
	189 (CardiologyUnit)	Not surprised	63	23	40	0.622 (0.448–0.775)	0.737 (0.659–0.805)	0.365 (0.285–0.453)	0.889 (0.840–0.924)	0.714 (0.644–0.778)
		Surprised	126	14	112					
Yen Y.-F.	21 098	Not surprised	2620	799	1821	0.456 (0.432–0.479)	0.906 (0.901–0.910)	0.305 (0.291–0.319)	0.948 (0.946–0.950)	0.868 (0.864–0.873)
		Surprised	18 478	955	17 523					
	193	Not surprised	103	44	59	0.786	0.569	0.427	0.867	0.632
AaronsonE.L.		Surprised	90	12	78	(0.656–0.884)	(0.482–0.654)	(0.371–0.486)	(0.794–0.916)	(0.560–0.700)
Schmidt R.J.	749	Not surprised	173	61	112	0.604 (0.502–0.700)	0.827 (0.796–0.856)	0.353 (0.302–0.407)	0.931 (0.913–0.945)	0.797 (0.766–0.825)
		Surprised	576	40	536					
Lakin J.R.	1448 (Nurses)	Not surprised	352	112	240	0.526 (0.457–0.595)	0.806 (0.783–0.827)	0.318 (0.282–0.356)	0.908 (0.895–0.919)	0.765 (0.742–0.786)
		Surprised	1096	101	995					
	1163 (Physicians)	Not surprised	452	143	309	0.794 (0.728–0.851)	0.686 (0.656–0.715)	0.316 (0.291–0.343)	0.948 (0.932–0.961)	0.703 (0.675–0.729)
		Surprised	711	37	674					
VeldhovenC.M.M.	292 (SQ1)	Not surprised	161	24	137	0.923 (0.749–0.991)	0.485 (0.424–0.547)	0.149 (0.130–0.171)	0.985 (0.944–0.996)	0.524 (0.465–0.583)
		Surprised	131	2	129					
	161 (SQ2)	Not surprised	22	10	12	0.417 (0.221–0.634)	0.912 (0.852–0.954)	0.455 (0.289–0.631)	0.899 (0.864–0.926)	0.830 (0.772–0.892)
		Surprised	139	14	125					
Burke K.	325 (majority vote– 90 days)	Not surprised	36	15	21	0.833 (0.586–0.964)	0.932 (0.897–0.957)	0.417 (0.310–0.531)	0.990 (0.971–0.996)	0.926 (0.892–0.952)
		Surprised	289	3	286					
	306 (majority vote– 365 days)	Not surprised	106	25	81	0.833 (0.653–0.944)	0.707 (0.649–0.760)	0.236 (0.195–0.282)	0.975 (0.946–0.989)	0.719 (0.665–0.769)
		Surprised	200	5	195					
	238 (100% agreement - 90 days)	Not surprised	21	14	7	0.933 (0.681–0.998)	0.969 (0.936–0.987)	0.667 (0.488–0.808)	0.995 (0.970–0.999)	0.966 (0.935–0.985)
		Surprised	217	1	216					
	175 (100% agreement – 365 days)	Not surprised	57	21	36	0.955 (0.772–0.999)	0.765 (0.689–0.829)	0.368 (0.302–0.441)	0.992 (0.945–0.999)	0.789 (0.721–0.847)
		Surprised	118	1	117					
	290 (75–100% agreement – 90 days)	Not surprised	26	14	12	0.824 (0.566–0.962)	0.956 (0.925–0.977)	0.539 (0.391–0.679)	0.989 (0.969–0.996)	0.948 (0.916–0.971)
		Surprised	264	3	261					
	236 (75–100% agreement – 365 days)	Not surprised	80	24	56	0.923 (0.749–0.991)	0.733 (0.668–0.792)	0.300 (0.250–0.355)	0.987 (0.953–0.997)	0.754 (0.694–0.808)
		Surprised	156	2	154					
	122 (Neurology –365 days)	Not surprised	28	7	21	0.875 (0.474–0.997)	0.816 (0.732–0.882)	0.250 (0.173–0.347)	0.989 (0.937–0.998)	0.820 (0.740–0.883)
		Surprised	94	1	93					
	27 (Oncology – 365 days)	Not surprised	22	12	10	1.000 (0.735–1.000)	0.333 (0.118–0.616)	0.546 (0.456–0.632)	1.000	0.630 (0.424–0.806)
		Surprised	5	0	5					
	73 (Congenital –365 days)	Not surprised	24	4	20	0.667 (0.223–0.957)	0.702 (0.577–0.807)	0.167 (0.093–0.282)	0.959 (0.882–0.987)	0.699 (0.580–0.801)
		Surprised	49	2	47					
Ouchi K.	207	Not surprised	102	34	68	0.773 (0.622–0.885)	0.583 (0.503–0.660)	0.333 (0.282–0.289)	0.905 (0.844–0.943)	0.623 (0.553–0.690)
		Surprised	105	10	95					
Liyanage T.	187	Not surprised	80	30	50	0.714 (0.554–0.843)	0.655 (0.572–0.732)	0.375 (0.309–0.446)	0.888 (0.829–0.928)	0.668 (0.596–0.735)
		Surprised	107	12	95					
Mitchell G.K.	2840 (Intuition)	Not surprised	154	32	122	0.337 (0.243–0.441)	0.956 (0.947–0.963)	0.208 (0.159–0.268)	0.977 (0.973–0.980)	0.935 (0.925–0.944)
		Surprised	2686	63	2623					
	1525 (ST)	Not surprised	179	25	154	0.532 (0.381–0.679)	0.896 (0.879–0.911)	0.140 (0.107–0.181)	0.984 (0.978–0.988)	0.885 (0.868–0.900)
		Surprised	1346	22	1324					
Ebke M.	236 (Neurorehabilitation Physicians)	Not surprised	45	17	28	0.500 (0.324–0.676)	0.861 (0.806–0.906)	0.378 (0.273–0.496)	0.911 (0.879–0.935)	0.809 (0.753–0.857)
		Surprised	191	17	174					
	236 (Palliative CarePhysicians)	Not surprised	83	23	60	0.677 (0.495–0.826)	0.703 (0.635–0.765)	0.277 (0.219–0.344)	0.928 (0.887–0.955)	0.699 (0.636–0.757)
		Surprised	153	11	142					
Mudge A.M.	100	Not surprised	52	16	36	0.889 (0.653–0.986)	0.561 (0.447–0.670)	0.308 (0.249–0.374)	0.958 (0.860–0.989)	0.620 (0.518–0.715)
		Surprised	48	2	46					
Salat H.	488	Not surprised	171	56	115	0.644 (0.534–0.744)	0.713 (0.666–0.757)	0.328 (0.281–0.378)	0.902 (0.874–0.925)	0.701 (0.658–0.741)
		Surprised	317	31	286					
Malhotra R.	208 (180 days)	Not surprised	203	10	193	0.769 (0.462–0.950)	0.010 (0.001–0.037)	0.049 (0.037–0.065)	0.400 (0.109–0.785)	0.058 (0.030–0.099)
		Surprised	5	3	2					
	189 (365 days)	Not surprised	162	13	149	0.650 (0.408–0.846)	0.118 (0.074–0.177)	0.080 (0.059–0.108)	0.741 (0.580–0.855)	0.175 (0.123–0.236)
		Surprised	27	7	20					
Hadique S.	500 (Derivation cohort)	Not surprised	238	148	90	0.822 (0.758–0.875)	0.719 (0.666–0.767)	0.622 (0.577–0.665)	0.878 (0.839–0.908)	0.756 (0.716–0.793)
		Surprised	262	32	230					
	543 (Validation cohort)	Not surprised	204	139	65	0.739 (0.671–0.801)	0.817 (0.773–0.856)	0.681 (0.628–0.730)	0.856 (0.822–0.883)	0.790 (0.753–0.824)
		Surprised	339	49	290					
Gomez- Batiste X.	1059 (365 days)	Not surprised	837	268	569	0.937 (0.902–0.962)	0.264 (0.233–0.297)	0.320 (0.309–0.332)	0.919 (0.877–0.947)	0.446 (0.416–0.476)
		Surprised	222	18	204					
	1059 (730 days)	Not surprised	837	373	464	0.914 (0.883–0.940)	0.287 (0.253–0.324)	0.446 (0.432–0.460)	0.842 (0.792–0.882)	0.529 (0.498–0.559)
		Surprised	222	35	187					
Javier A.D.	388	Not surprised	137	33	104	0.635 (0.490–0.764)	0.691 (0.638–0.740)	0.241 (0.196–0.292)	0.924 (0.894–0.946)	0.683 (0.634–0.729)
		Surprised	251	19	232					
Amro O.W.	201	Not surprised	50	22	28	0.550 (0.385–0.707)	0.826 (0.759–0.881)	0.440 (0.336–0.549)	0.881 (0.839–0.913)	0.771 (0.707–0.827)
		Surprised	151	18	133					
Lakin J.R.	1737	Not surprised	114	23	91	0.205 (0.135–0.292)	0.944 (0.932–0.955)	0.202 (0.143–0.277)	0.945 (0.940–0.950)	0.896 (0.881–0.910)
		Surprised	1623	89	1534					
Hamano J.	2361 (7 days)	Not surprised	931	282	649	0.847 (0.804–0.884)	0.680 (0.659–0.700)	0.303 (0.287–0.320)	0.964 (0.955–0.972)	0.704 (0.685–0.722)
		Surprised	1430	51	1379					
	2361 (30 days)	Not surprised	1851	1066	785	0.956 (0.942–0.967)	0.370 (0.343–0.397)	0.576 (0.565–0.587)	0.904 (0.876–0.926)	0.647 (0.627–0.666)
		Surprised	510	49	461					
Feyi K.	178	Not surprised	58	37	21	0.726 (0.583–0.841)	0.835 (0.758–0.895)	0.638 (0.535–0.730)	0.883 (0.828–0.923)	0.803 (0.737–0.859)
		Surprised	120	14	106					
Moroni M.	231	Not surprised	126	87	39	0.837 (0.751–0.902)	0.693 (0.605–0.772)	0.691 (0.629–0.746)	0.838 (0.768–0.890)	0.758 (0.697–0.811)
		Surprised	105	17	88					
O'CallaghanA.	501 (180 days)	Not surprised	99	56	43	0.727 (0.614–0.823)	0.899 (0.866–0.926)	0.566 (0.487–0.641)	0.948 (0.926–0.963)	0.872 (0.840–0.900)
		Surprised	402	21	381					
	501 (365 days)	Not surprised	99	67	32	0.626 (0.527–0.718)	0.919 (0.887–0.944)	0.677 (0.593–0.751)	0.901 (0.876–0.920)	0.856 (0.823–0.886)
		Surprised	402	40	362					
Da SilvaGane M.	3896	Not surprised	938	281	657	0.496 (0.455–0.538)	0.803 (0.789–0.816)	0.300 (0.278–0.323)	0.904 (0.896–0.911)	0.758 (0.744–0.772)
		Surprised	2958	285	2673					
Pang W.-F.	367	Not surprised	109	27	82	0.614 (0.455–0.756)	0.746 (0.695–0.793)	0.248 (0.196–0.308)	0.934 (0.907–0.9540	0.730 (0.682–0.775)
		Surprised	258	17	241					
Haga K.	138	Not surprised	120	39	81	0.886 (0.754–0.962)	0.138 (0.076–0.225)	0.325 (0.297–0.355)	0.722 (0.497–0.873)	0.377 (0.296–0.463)
		Surprised	18	5	13					
Fenning S.	172	Not surprised	38	6	32	0.353 (0.142–0.617)	0.794 (0.721–0.854)	0.158 (0.084–0.277)	0.918 (0.886–0.941)	0.750 (0.678–0.813)
		Surprised	134	11	123					
Moss A.H.	826	Not surprised	131	53	78	0.747 (0.629–0.842)	0.897 (0.873–0.918)	0.405 (0.346–0.466)	0.974 (0.962–0.983)	0.884 (0.860–0.905)
		Surprised	695	18	677					
Cohen L.M.	450	Not surprised	71	39	32	0.379 (0.285–0.480)	0.908 (0.872–0.936)	0.549 (0.447–0.648)	0.831 (0.808–0.852)	0.787 (0.746–0.824)
		Surprised	379	64	315					
Moss A.H.	147	Not surprised	34	10	24	0.455 (0.244–0.678)	0.808 (0.728–0.873)	0.294 (0.189–0.427)	0.894 (0.851–0.926)	0.755 (0.677–0.822)
		Surprised	113	12	101					
Ros M.M.	3140 (ICU stay)	Not surprised	153	98	55	0.363 (0.306–0.423)	0.981 (0.975–0.986)	0.641 (0.568–0.708)	0.942 (0.937–0.947)	0.928 (0.918–0.937)
		Surprised	2987	172	2815					
	3140 (Hospital stay)	Not surprised	252	148	104	0.378 (0.329–0.428)	0.962 (0.954–0.969)	0.587 (0.531–0.641)	0.916 (0.909–0.921)	0.889 (0.878–0.900)
		Surprised	2888	244	2644					
	3140 (365 days)	Not surprised	609	363	246	0.509 (0.471–0.546)	0.899 (0.886–0.910)	0.596 (0.562–0.629)	0.862 (0.852–0.871)	0.810 (0.796–0.824)
		Surprised	2531	350	2181					
GuliniJ.E.H.M.B	170	Not surprised	89	41	48	0.820 (0.686–0.914)	0.600 (0.507–0.688)	0.461 (0.398–0.524)	0.889 (0.813–0.936)	0.665 (0.588–0.7350
		Surprised	81	9	72					
Haydar S.A.	6122	Not surprised	918	107	811	0.682 (0.603–0.754)	0.864 (0.855–0.873)	0.117 (0.104–0.130)	0.990 (0.988–0.992)	0.859 (0.850–0.868)
		Surprised	5204	50	5154					
Carmen J.	49	Not surprised	20	7	13	0.778 (0.400–0.972)	0.675 (0.509–0.814)	0.350 (0.234–0.487)	0.931 (0.796–0.979)	0.694 (0.546–0.818)
		Surprised	29	2	27					
Barnes S.	231	Not surprised	95	11	84	0.786 (0.492–0.953)	0.613 (0.545–0.678)	0.116 (0.087–0.153)	0.978 (0.942–0.992)	0.623 (0.558–0.686)
		Surprised	136	3	133					
Straw S.	114 (Consultants)	Not surprised	64	33	31	0.846 (0.695–0.941)	0.587 (0.467–0.699)	0.516 (0.441–0.590)	0.880 (0.774–0.940)	0.675 (0.581–0.760)
		Surprised	50	6	44					
	128 (JuniorDoctors)	Not surprised	65	33	32	0.750 (0.597–0.868)	0.619 (0.507–0.723)	0.508 (0.428–0.587)	0.825 (0.734–0.890)	0.664 (0.575–0.745)
		Surprised	63	11	52					
	89 (Heart FailureNurses)	Not surprised	60	27	33	0.900 (0.735–0.979)	0.441 (0.312–0.576)	0.450 (0.388–0.514)	0.897 (0.740–0.963)	0.596 (0.496–0.698)
		Surprised	29	3	26					
	123 (Staff Nurses)	Not surprised	50	29	21	0.659 (0.501–0.795)	0.734 (0.623–0.827)	0.580 (0.475–0.678)	0.795 (0.715–0.856)	0.707 (0.619–0.786)
		Surprised	73	15	58					
	119 (2 or more)	Not surprised	62	26	36	0.818	0.577	0.500	0.860	0.659
		Surprised	57	8	49	(0.673–0.918)	(0.465–0.683)	(0.429–0.571)	(0.761–0.922)	(0.570–0.740)
	129 (3 or more)	Not surprised	54	31	23	0.705 (0.548–0.832)	0.729 (0.622–0.820)	0.574 (0.475–0.667)	0.827 (0.748–0.885)	0.721 (0.635–0.796)
		Surprised	75	13	62					
	129 (4 ‘no’s’)	Not surprised	18	14	4	0.318 (0.186–0.476)	0.953 (0.884–0.987)	0.778 (0.551–0.909)	0.730 (0.687–0.769)	0.736 (0.652–0.810)
		Surprised	111	30	81					
	129 (all ‘no’)	Not surprised	35	23	12	0.523 (0.367–0.675)	0.859 (0.766–0.925)	0.657 (0.514–0.777)	0.777 (0.716–0.827)	0.744 (0.660–0.817)
		Surprised	94	21	73					
	32 (Diabetes)	Not surprised	20	9	11	0.900 (0.555–0.998)	0.500 (0.282–0.718)	0.450 (0.339–0.566)	0.917 (0.621–0.987)	0.625 (0.437–0.789)
		Surprised	12	1	11					
	82 (no diabetes)	Not surprised	44	24	20	0.828 (0.642–0.942)	0.623 (0.479–0.752)	0.546 (0.450–0.638)	0.868 (0.743–0.938)	0.695 (0.584–0.792)
		Surprised	38	5	33					
	35 (IHD)	Not surprised	20	14	6	0.824 (0.566–0.962)	0.667 (0.450–0.867)	0.700 (0.539–0.823)	0.800 (0.577–0.922)	0.743 (0.567–0.875)
		Surprised	15	3	12					
	79 (no IHD)	Not surprised	44	19	25	0.864 (0.651–0.971)	0.562 (0.424–0.693)	0.432 (0.352–0.516)	0.914 (0.784–0.969)	0.646 (0.530–0.750)
		Surprised	35	3	32					
	18 (eGFR<30)	Not surprised	17	10	7	0.909 (0.587–0.998)	0.000 (0.000–0.410)	0.588 (0.542–0.633)	0.000	0.556 (0.308–0.785)
		Surprised	1	1	0					
	96 (eGFR>30)	Not surprised	47	23	24	0.821 (0.631–0.939)	0.647 (0.522–0.759)	0.489 (0.399–0.580)	0.898 (0.796–0.952)	0.698 (0.596–0.788)
		Surprised	49	5	44					
Mahes A.	301	Not surprised	136	25	111	0.807 (0.625–0.926)	0.589 (0.528–0.648)	0.184 (0.152–0.220)	0.964 (0.928–0.982)	0.611 (0.554–0.667)
		Surprised	165	6	159					
Lin C.A.	840	Not surprised	214	32	182	0.615 (0.483–0.748)	0.768 (0.739–0.798)	0.149 (0.101–0.196)	0.968 (0.954–0.982)	0.759 (0.730–0.788)
		Surprised	626	20	606					
Um Y.W.	300	Not surprised	118	25	93	0.833 (0.653–0.944)	0.662 (0.603–0.718)	0.212 (0.176–0.253)	0.973 (0.942–0.988)	0.679 (0.623–0.731)
		Surprised	182	5	177					
Gaffney L.	191	Not surprised	56	20	36	0.571 (0.394–0.737)	0.769 (0.695–0.833)	0.357 (0.270–0.455)	0.889 (0.844–0.922)	0.733 (0.664–0.794)
		Surprised	135	15	120					

Sensitivity (the ability of the prompt to successfully identify those patients who were dying); specificity (the ability of the prompt to successfully identify those who were not dying); positive predictive value (the proportion of patients who died when the respondent predicted death); negative predictive value (the proportion of the patients who survived when the respondent predicted survival); accuracy (the proportion of correct predictions among all cases).

### Accuracy according to event rates

The overall mortality rates ranged from 2.6% to 81.5%. We divided studies into three groups to explore how the accuracy of the Surprise Question varied by mortality rate. Nineteen studies reported <14% of their patients dying. In these studies, respondents identified in 62% of cases those that died (sensitivity 0.62 (0.53 to 0.72) I^2^=96.3%) and those that did not in 74% of cases (specificity 0.74 (0.64 to 0.83) I^2^=99.9%). Eighteen studies reported a mortality rate of 14%–23%. In these studies, respondents successfully predicted death in 61% of cases (sensitivity 0.61 (0.55 to 0.66) I^2^=84.3%) and survival in 77% of cases (specificity 0.77 (0.72 to 0.81) I^2^=97.0%). More than 23% of patients died in the remaining 19 studies. In these studies, respondents performed best when identifying patients who were likely to die (sensitivity 0.83 ((0.79 to 0.87) I^2^=92.3%). They successfully identified those that would not die in 56% of cases (specificity 0.56 (0.46 to 0.65) I^2^=98.4%).

Where respondents predicted death, the proportion of patients that actually died was greatest in those studies with an event rate >23% (PPV 0.58 [0.49 to 0.66] I^2^=99.4%) and lowest in those with an event rate<14% (PPV 0.22 [0.18 to 0.27] I^2^=98.0). Conversely, when respondents predicted survival, the proportion of patients that survived was greatest in studies with lower event rates (NPV 0.96 (0.95 to 0.97) I^2^=98.3) and lowest in those studies with larger event rates (0.80 (0.75 to 0.86) I^2^=96.9).

### Accuracy according to setting

**Figure 2:**
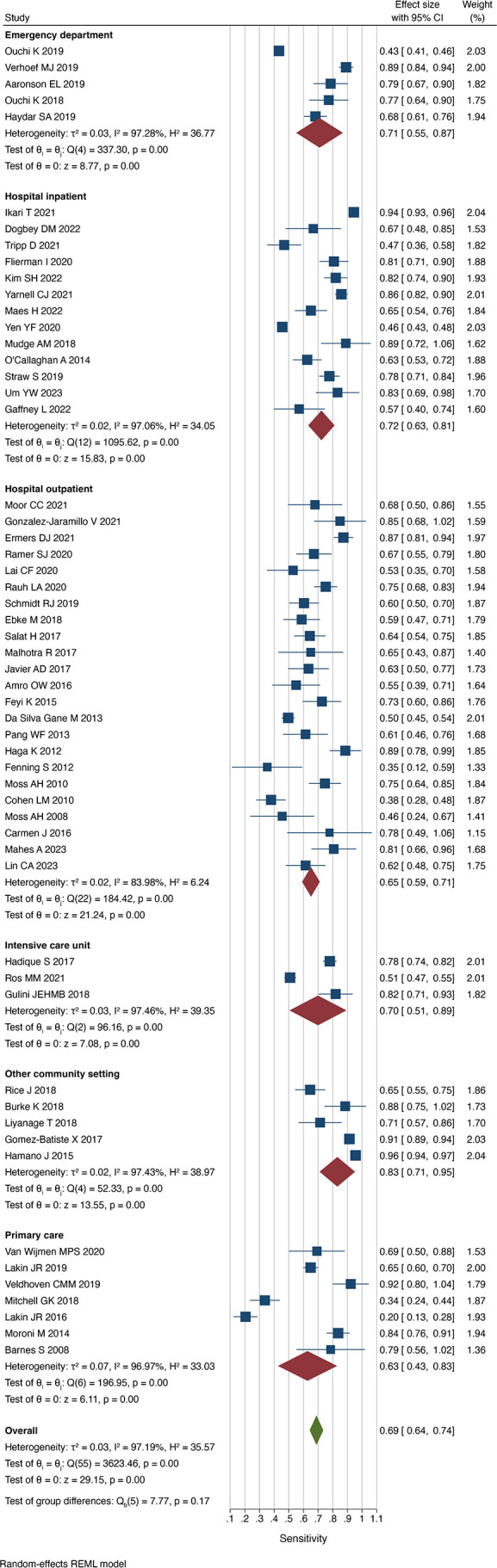
Sensitivity of the Surprise Question by setting. REML, restricted maximum-likelihood.

**Figure 3:**
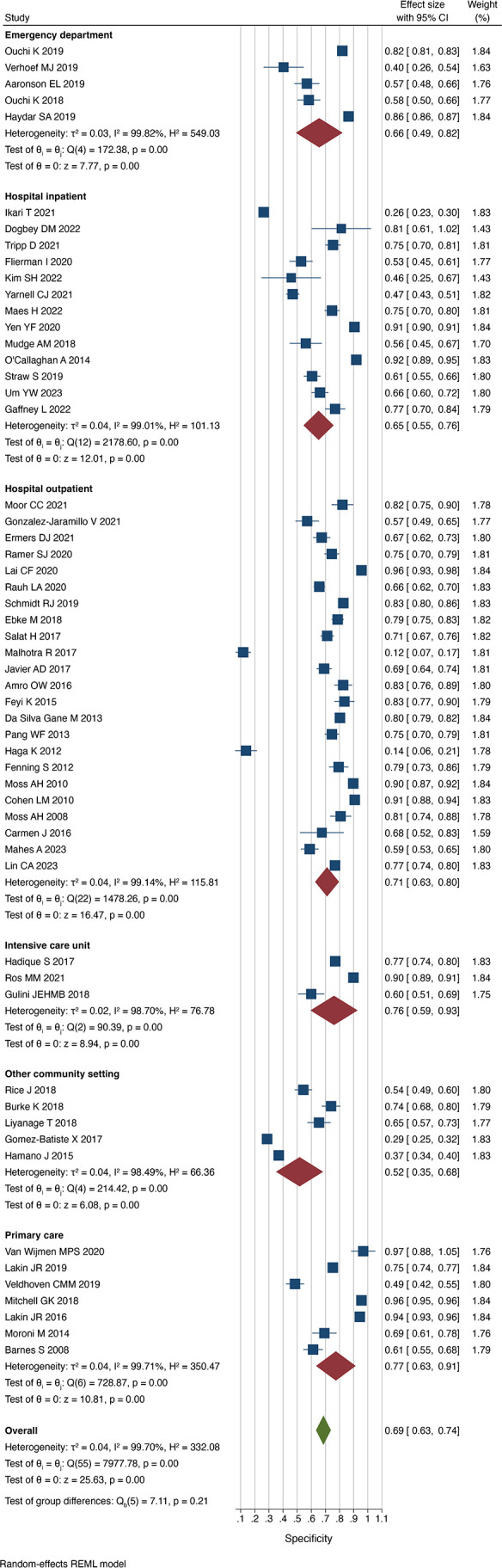
Specificity of the Surprise Question by setting. REML, restricted maximum-likelihood.

Respondents were most reliably able to identify those patients at risk of dying within the follow-up time in community settings (sensitivity 0.83 (0.71 to 0.95), I^2^=97.4%), whereas in studies performed in primary care settings, the sensitivity was lowest (0.63 (0.43 to 0.83)], I^2^=97.0%) (figure 2). Conversely, respondents in studies set in primary care were most successful in being able to identify those that would survive (specificity 0.77 (0.63 to 0.91), I^2^=99.7%), whereas respondents in community care settings including nursing homes and hospices were least able to identify those that survived (specificity 0.52 (0.35 to 0.68), I^2^=98.5%) (figure 3). When respondents predicted death, this was correct most commonly in intensive care units at (PPV 0.57 (0.46 to 0.68), I^2^=95.2%) and incorrect most commonly in the outpatient setting (0.34 (0.28 to 0.40) I^2^=97.1%). When respondents predicted survival, this was most commonly the case in primary care patients (NPV 0.93 (0.88 to 0.98), I^2^=99.4%) and incorrect most commonly for hospital inpatients (NPV 0.83 (0.75 to 0.91), I^2^=99.2%).

### Accuracy according to specialty

**Figure 4:**
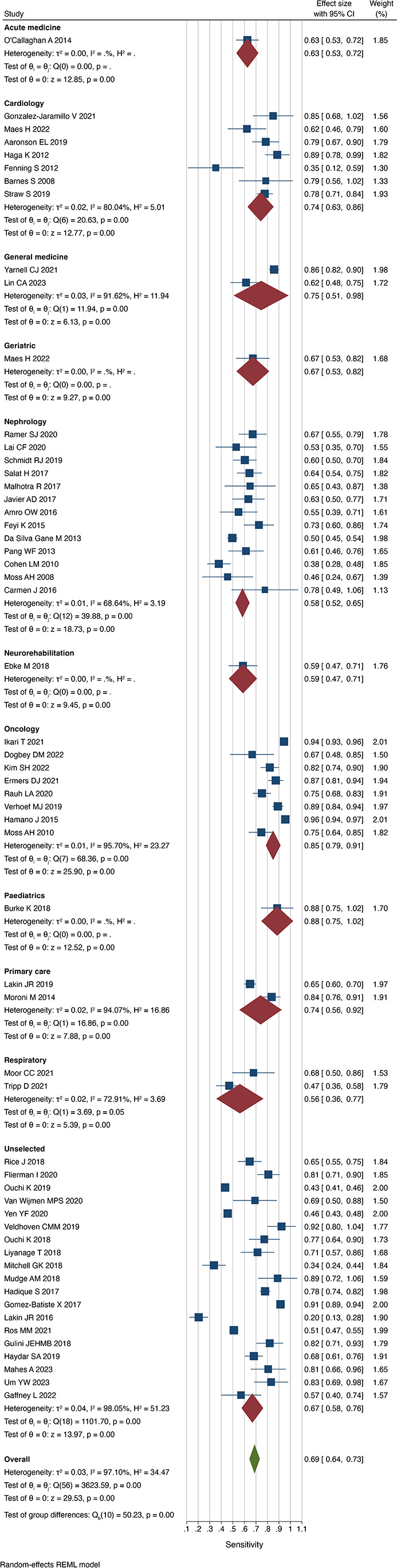
Sensitivity of the Surprise Question by specialty. REML, restricted maximum-likelihood.

**Figure 5:**
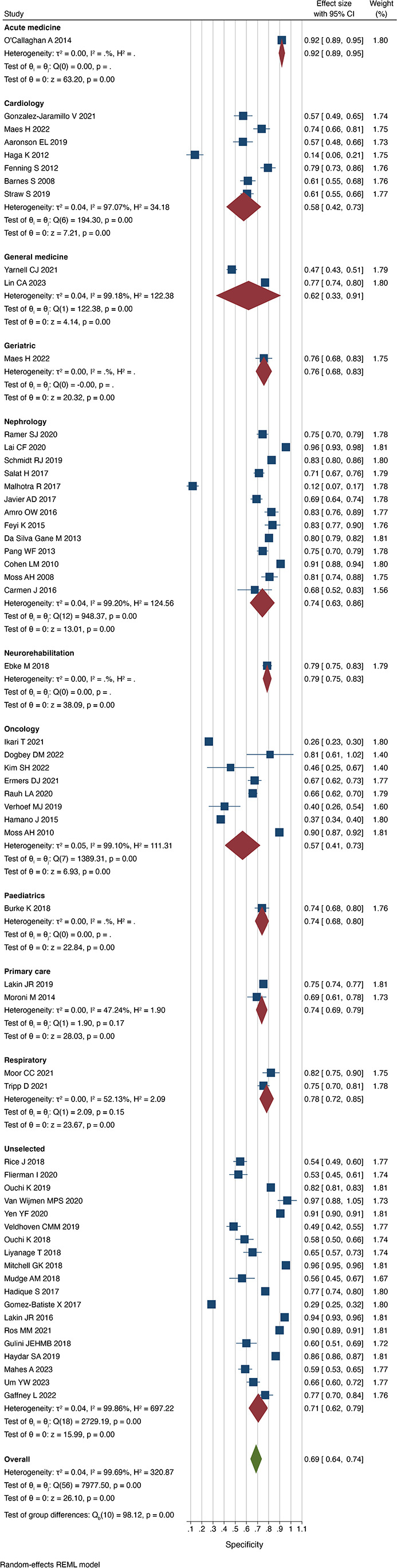
Specificity of the Surprise Question by specialty. REML, restricted maximum-likelihood.

We observed significant heterogeneity in the performance of the Surprise Question according to specialty. Respondents were most successful at predicting death in the paediatric cohort (sensitivity 0.88 (0.75 to 1.02)) and were incorrect mostly in respiratory cohorts (sensitivity 0.56 (0.36 to 0.77) I^2^=72.9%) (figure 4). The Surprise Question performed best in acute medical patients when identifying those that were not at risk of dying (specificity 0.92 (0.89 to 0.95)) and worst in oncology patients (0.57 (0.41 to 0.73) I^2^=99.1%) (figure 5). The proportion of patients who died when the respondent predicted death ranged from 30% (PPV 0.30 (0.19 to 0.41) I^2^=97.6%) in cardiology patients to 68% (PPV 0.68 (0.60 to 0.76)) in acute medical patients. The proportion of patients who survived when the respondent predicted survival was generally consistent across all specialties but was lowest in oncological patients with a value of 76% (NPV 0.76 (0.60 to 0.91) I^2^=99.5%). The accuracy was greatest in acute medical patients (0.86 (0.82 to 0.89)) and lowest in cardiology and general medical patients (0.63 (0.54 to 0.71) I^2^=91.9% and 0.69 (0.55 to 0.83) I^2^=97.4%, respectively).

### Accuracy according to follow-up period

Thirteen studies assessed the performance of the Surprise Question over time periods shorter than 1 year.^12^,^24^ In these studies, the prompt successfully predicted death in 69% of cases (sensitivity 0.69 (0.56 to 0.82) I^2^=99.3%) and successfully predicted survival in 65% (specificity 0.65 ((0.49 to 0.81) I^2^=99.9%). The proportion of patients who died when the respondent predicted death was greatest in this subgroup (PPV 0.44 (0.29 to 0.59) I^2^=99.9%), compared with at 1 year (PPV 0.38 (0.32 to 0.44) I^2^=99.0%) and over 1 year (PPV 0.36 (0.30 to 0.41) I^2^=93.4%).

Fourty-two studies assessed the prognostic accuracy of the Surprise Question at 1 year.^714 17 20 25^,^62^ In a pooled comparison, the proportion of correct predictions among all cases was 71% (accuracy 0.71 (0.67 to 0.75) I^2^=99.2%). The sensitivity of a ‘not surprised’ response was 0.68 ((0.63 to 0.74) I^2^=95.0%) and the specificity was 0.69 ((0.63 to 0.75) I^2^=99.7%).

**Figure 6:**
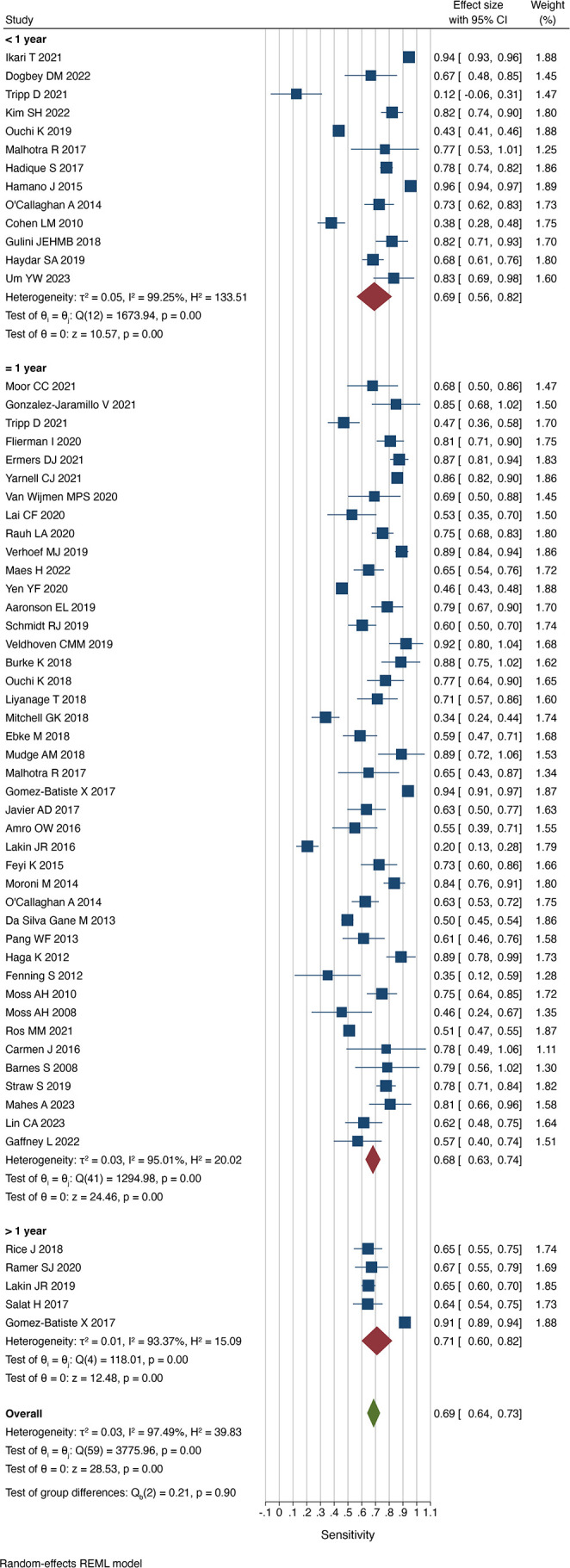
Sensitivity of the Surprise Question by timeframe. REML, restricted maximum-likelihood.

Five studies used a timeframe of greater than 1 year.^2550 63^,^66^ Respondents successfully identified those at risk of dying and those surviving in 71% (sensitivity 0.71 (0.60 to 0.82) I^2^=93.4%) and 61% (specificity 0.61 (0.43 to 0.78) I^2^=99.1%), respectively (figures6  7). This was relatively comparable to the other subgroups.

**Figure 7:**
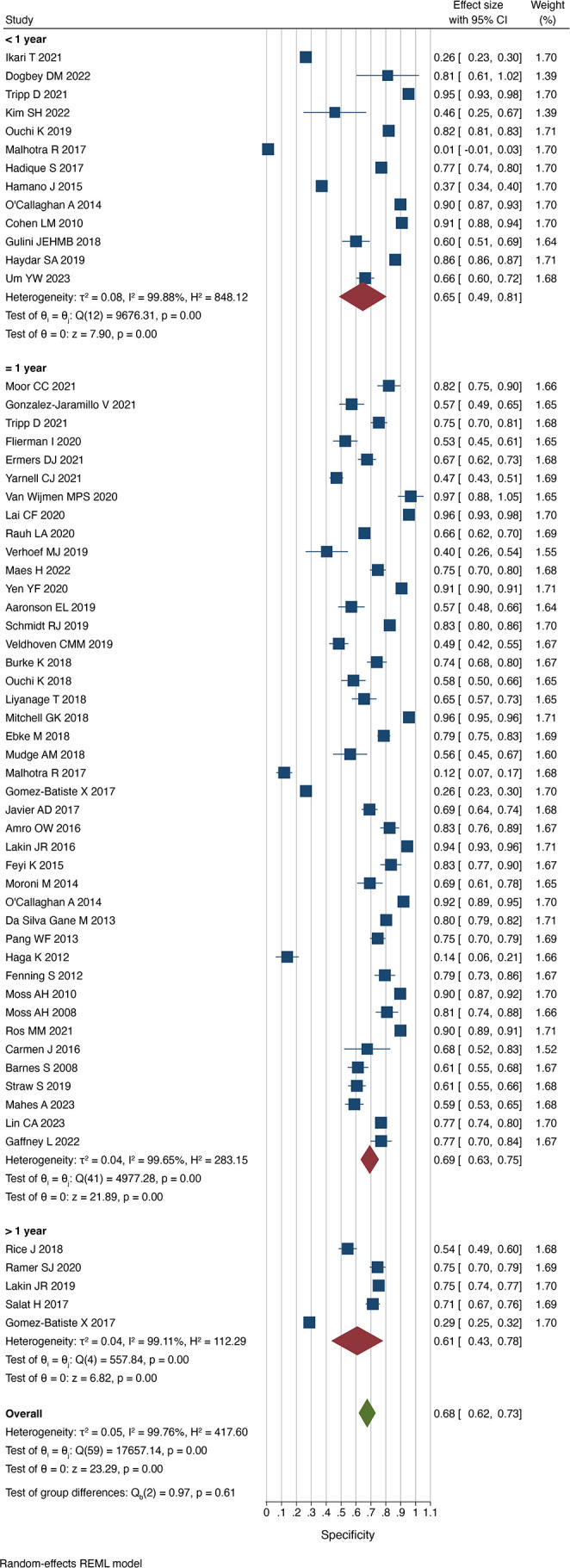
Specificity of the Surprise Question by timeframe. REML, restricted maximum-likelihood.

### Accuracy according to respondent

**Figure 8:**
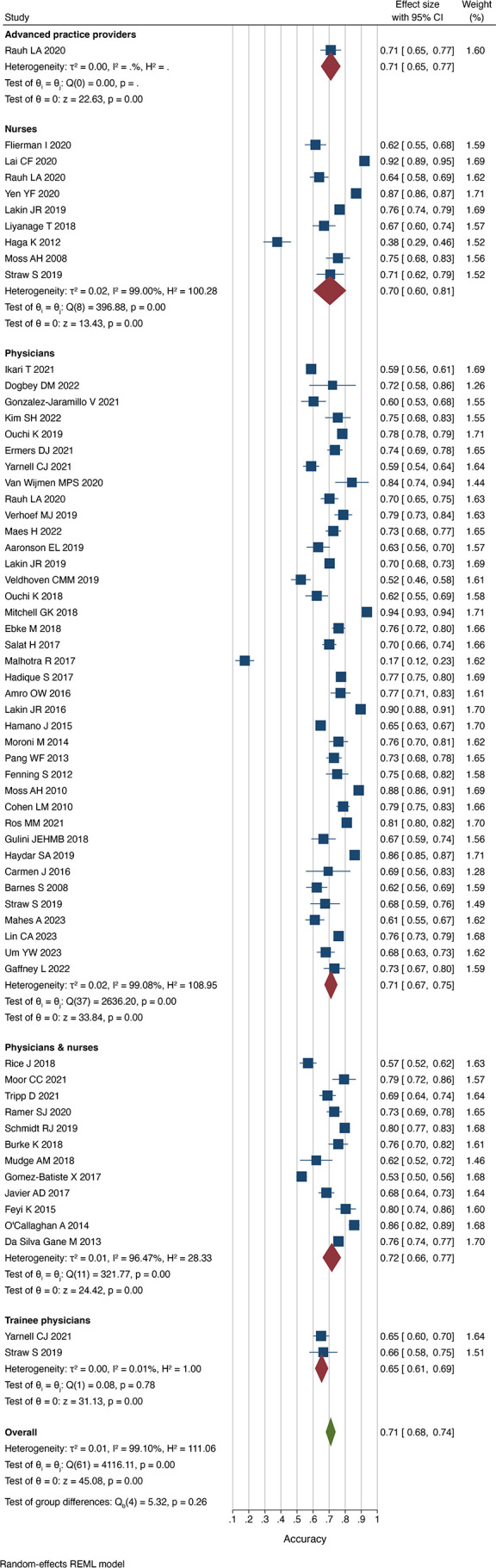
Accuracy of the Surprise Question by respondent. REML, restricted maximum-likelihood.

Four studies reported responses to the Surprise Question from different healthcare professionals.^7 37 42 63^ Studies, where physicians and nurses together provided responses to the Surprise Question, resulted in the highest proportion of patients successfully identified at risk of dying (sensitivity 0.71 (0.61 to 0.81) I^2^=98.7%). Trainee physicians performed worst in identifying those that did not die within the follow-up period (0.57 (0.49 to 0.64) I^2^=33.4%). Of those predicted to die within the follow-up period by nurses, 39% of them did die (PPV 0.39 (0.33 to 0.45) I^2^=94.3%), compared with 52% when trainee physicians predicted death (PPV 0.52 (0.49 to 0.56) I^2^=0.0%). Conversely, 83% of patients survived when trainee physicians predicted survival (NPV 0.83 (0.79 to 0.87) I^2^=0.0%), compared with 93% in advanced practice providers (NPV 0.93 (0.88 to 0.97)). The pooled accuracy was similar between physicians (0.71 (0.67 to 0.75) I^2^=99.1%) and nurses (0.70 (0.60 to 0.81) I^2^=99.0%) (figure 8).

### Risk of bias of included studies

Full details of the risk of bias assessment are displayed in table 4. Overall, 41 studies were rated ‘good’ quality, with the remaining 15 studies being rated as ‘poor’ quality. The most common reasons for bias were failure to control for age and/or sex (n=14, 25.0%), failure to control for any other additional factors (n=13, 23.2%) or the method for ascertainment of outcomes not being documented (n=10, 17.9%). However, given the aim of the current study, study quality is unlikely to have had a significant impact on our analysis.

**Table 4:** Newcastle-Ottawa scale for included studies

First author	Representative ness ofexposed cohort	Selection of non-exposedcohort	Ascertainment of exposure	Outcome of interestaccounted for	Age/gender	Other factors	Ascertainment of outcomes	Length of follow-up	Adequacy of follow-up	Quality of study
Rice J.	*	*	*	*	*		*	*	*	Good
Ikari T.	*	*	*	*	*	*		*	*	Good
Dogbey D.M.	*	*	*	*	*	*	*	*	*	Good
Moor C.C.	*	*	*	*	*	*	*	*	*	Good
Gonzalez-Jaramillo V.	*	*	*	*	*	*	*	*	*	Good
Tripp D.	*	*	*	*	*	*	*	*	*	Good
Flierman I.	*	*	*	*			*	*	*	Poor
Kim S.H.		*	*	*		*		*	*	Good
Ouchi K.	*	*	*	*	*	*	*	*		Good
Ermers D.J.M.	*	*	*	*			*	*	*	Poor
Yarnell C.	*	*	*	*	*	*	*	*	*	Good
Van WijmenM.P.S.	*	*	*	*	*	*	*	*	*	Good
Ramer S.J.	*	*	*	*	*	*	*	*	*	Good
Lai C.-F.		*	*	*	*	*			*	Poor
Rauh L.A.	*	*	*	*	*	*	*	*	*	Good
Verhoef M.J.	*	*	*	*			*	*	*	Poor
Maes H.	*	*	*	*	*	*	*	*	*	Good
Yen Y.-F.	*	*	*	*	*	*	*		*	Good
Aaronson E.L.	*	*	*	*		*	*		*	Good
Schmidt R.J.	*	*	*	*	*	*	*	*	*	Good
Lakin J.R.	*	*	*	*	*	*	*	*	*	Good
VeldhovenC.M.M.	*	*	*	*			*		*	Poor
Burke K.	*	*	*	*	*	*	*		*	Good
Ouchi K.	*	*	*	*			*	*	*	Poor
Liyanage T.	*	*	*	*			*	*	*	Poor
Mitchell G.K.	*	*	*	*			*		*	Poor
Ebke M.	*	*	*	*	*	*		*	*	Good
Mudge A.M.	*	*	*	*			*	*	*	Poor
Salat H.	*	*	*	*	*	*	*	*	*	Good
Malhotra R.	*	*	*	*				*	*	Poor
Hadique S.	*	*	*	*	*	*	*	*	*	Good
Gomez-BatisteX.	*	*	*	*	*	*	*	*	*	Good
Javier A.D.	*	*	*	*	*	*	*	*	*	Good
Amro O.W.	*	*	*	*	*	*	*	*	*	Good
Lakin J.R.	*	*	*	*	*	*	*		*	Good
Hamano J.		*	*	*	*	*		*	*	Good
Feyi K.	*	*	*	*	*	*		*	*	Good
Moroni M.	*	*	*	*	*	*	*	*	*	Good
O'Callaghan A.	*	*	*	*	*	*	*	*	*	Good
Da Silva GaneM.	*	*	*	*	*	*	*	*	*	Good
Pang W.-F.	*	*	*	*			*	*	*	Poor
Haga K.	*	*	*	*			*	*	*	Poor
Fenning S.	*	*	*	*	*	*	*	*		Good
Moss A.H.	*	*	*	*	*	*	*	*	*	Good
Cohen L.M.	*	*	*	*	*	*		*	*	Good
Moss A.H.	*	*	*	*	*	*		*	*	Good
Ros M.M.	*	*	*	*	*	*	*	*	*	Good
GuliniJ.E.H.M.B.	*	*	*	*	*	*	*	*	*	Good
Haydar S.A.	*	*	*	*	*	*	*			Poor
Carmen J.	*	*	*	*				*	*	Poor
Barnes S.	*	*	*	*	*	*	*			Poor
Straw S.	*	*	*	*	*	*	*	*	*	Good
Mahes A.	*	*	*	*	*	*	*	*	*	Good
Lin C.A.	*	*	*	*	*	*	*	*	*	Good
Um Y.W.	*	*		*	*	*	*	*	*	Good
Gaffney L.	*	*	*	*	*	*	*	*	*	Good

* denotes a study having met a particular quality item, scoring one point. The maximum points a study can score is nine, and the minimum is zero.

## Discussion

In this meta-analysis, the accuracy of the Surprise Question was assessed across a diverse range of studies including a total of 68 829 unique patients. In the overall pooled comparison, the accuracy of the Surprise Question was modest, in keeping with prior meta-analyses.^8 9^ We found its performance varied considerably according to the event rate of the population in which the prompt was applied, the healthcare setting, specialty, follow-up period chosen, and to whom the Surprise Question was posed.

We found that in studies where a greater proportion of the cohort died, clinicians were more reliably able to recognise this, in keeping with previous findings.^67^ One possible explanation is that where death is common, healthcare providers may become more realistic regarding patient prognosis, or more cognisant of the known predictors of poor outcomes for these patient groups. Prior meta-analyses have shown the Surprise Question to be more accurate in the setting of oncology compared with other disease groups.^8^,^10^ In our study, the ability of the Surprise Question to successfully identify those patients who were dying in oncology settings was excellent (PPV 0.85 (0.79 to 0.91) I^2^=95.7%), and higher than most other disease groups with the exception of the paediatric cohort (PPV 0.88 (0.75 to 1.02)), for which it was similar. It may be the case that patients diagnosed with malignancies exhibit a more predictable and consistent disease progression compared with those with other chronic conditions such as heart failure or chronic respiratory disease, where disease trajectories often display greater variability and unpredictability.^68^

When studies were divided by timeframe, the rate of identifying patients that were dying were similar (sensitivity <1 year=0.69 (0.56 to 0.82) I^2^=99.3%; 1 year=0.68 (0.63 to 0.74) I^2^=95.0%; >1 year=0.71 (0.60 to 0.82) I^2^=93.4%). A prior meta-analysis found that there were no differences in the accuracy of the Surprise Question when study timeframes shorter than 1 year were included, although in a limited sample of studies.^9^ The ability of the prompt to identify those that were not at risk of death was lower for timeframes above 1 year (specificity 0.61 (0.43 to 0.78) I^2^=99.1%) compared with 1 year and <1 year (specificity 0.69 (0.63 to 0.75) I^2^=99.7% and 0.65 (0.49 to 0.81) I^2^=99.9%, respectively). The reduced specificity for timeframes exceeding 1 year implies its potential inaccuracy for identifying patients unlikely to die over longer periods. This may raise concerns about overestimating the need for end-of-life care, potentially leading to unnecessary interventions for patients not in immediate need. Similar challenges have been observed in other prognostication models, highlighting the importance of cautious interpretation and further refinement in predicting longer term outcomes.^69^ Patients’ health conditions and anticipated prognoses may change over time, leading to uncertainties in predicting their need for end-of-life care. Additionally, healthcare providers may find it more challenging to accurately assess and predict patients’ needs for end-of-life care further into the future, as it involves a greater degree of uncertainty and more comprehensive assessments.

We found that trainee physicians performed comparatively worse compared with qualified physicians when identifying those that are unlikely to die, in line with other data suggesting that more experienced assessors are more accurate.^70^,^72^ A study of paediatrician’s survival predictions for premature new-born babies investigated whether physician’s self-rated attitude of being an optimist or a pessimist affected prediction accuracy. This study found that those physicians who rated themselves as optimistic, produced survival estimates which were accurate and comparable to true survival rates, while pessimists’ estimates consistently underestimated true survival rates.^73^ A further study of neonatologists in Italy concurred.^74^ This discrepancy may stem from the tendency of junior physicians to harbour more pessimistic attitudes, potentially affecting their predictive accuracy when compared with their senior counterparts, who tend to be less pessimistic and more precise in their assessments.

The Surprise Question is a core component of the Gold Standards Framework tool in the United Kingdom, which is recommended for use across primary and secondary healthcare settings to identify those nearing the end-of-life.^4^ Additionally, the Surprise Question has recently been endorsed in position statements by both the American Heart Association^5^ and Japanese Cardiology Society/Heart Failure Society.^6^ Recently, the Centre to Advance Palliative Care convened a consensus panel, which recommended that a ‘not surprised’ response to the Surprise Question should trigger assessment for unmet palliative care needs.^67^

While the Surprise Question is becoming more widespread and is widely endorsed, there are important practical considerations. One limitation lies in its reliance on the subjective judgement of healthcare practitioners, whose prognostic assessments may vary based on individual experiences and perceptions.^75^ One way of addressing this is by attempting to reach consensus. One study looked at the performance of the Surprise Question when utilised by a multidisciplinary team. When compared with a consensus that was restricted to either 100% or 75%–100% agreement among the multidisciplinary team, the analyses demonstrated that using a consensus opinion did result in a slightly lower overall accuracy, yet it did not significantly affect the prognostication results.^26^ A further study analysed the agreement of responses to the Surprise Question between different healthcare professionals for patients with heart failure. The study found the greatest agreement to be between cardiologists and heart failure nurse specialists, perhaps reflecting greater expertise and experience for these healthcare professionals compared with non-specialists.^7^ A further consideration is that the Surprise Question tends to result in an over classification of patients as ‘not surprised’. The Surprise Question could, therefore, be a valuable prognostic tool to identify those unlikely to die, and as a prompt to consider advanced care planning and referral to specialist palliative care services in populations where a nocebo effect from palliative care interventions is not considered likely.

A high false-positive rate may not be necessarily viewed as detrimental to patient care, as this may encourage clinicians to consider an early integration of palliative care into the patient pathway for those in whom death is possible, however it may have implications for service delivery. A holistic patient assessment is integral in the decision to refer to palliative care services, as opposed to a prognostic estimate alone, which is only one consideration. The possibility of a nocebo effect may be a concern to some, however a palliative approach is unlikely to be detrimental to patient outcomes where it is implemented alongside usual care and is complimentary to it.^68^ Therefore, the Surprise Question may be useful for identifying patients who may benefit from an early integration of palliative care, it should not be used as the sole determinant of treatment decisions.

### Strengths and limitations

Our data have several strengths over previous meta-analyses investigating the accuracy of the Surprise Question. Foremost, we include additional studies due to utilising a broad search strategy, including articles published or in press by 1 January 2024 as well as making requests to corresponding authors for unpublished data. Second, our analysis offers insights across a spectrum of healthcare settings, populations, follow-up intervals, respondents and event rates. Furthermore, each stage of the review process was conducted independently by two reviewers and the study protocol was registered prospectively.

Some limitations should be noted. First, in 12 studies, the respondents to the Surprise Question were ‘physicians and nurses’^14 20 26 35 38 44 47 49 50 52 64 65^ and data were not available to separately calculate the accuracy of each healthcare professional. However, it should be noted that there is evidence to suggest that multiprofessional predictions on prognosis are more accurate than single-professional estimates.^26 76^ Second, 26 studies were excluded after full-text review due to unavailability of raw data following requests to corresponding authors, which may result in sample bias.

## Conclusions

Our meta-analysis helps define the potential role of the Surprise Question as a prognostic tool in acute and chronic illness. We found that the overall accuracy of Surprise Question was modest, and that it performs best in populations where death is common, when posed over a shorter follow-up period, and to more experienced respondents. Despite its limitations, it may be the case that when considering supportive care, prognostication is less important, as those patients identified by the Surprise Question who do not subsequently die may still benefit from an early integration of a palliative approach into their care. Future studies should address whether integrating the Surprise Question into routine clinical care improves access to palliative care services, facilitates advance care planning and is acceptable to the healthcare team.

## supplementary material

10.1136/spcare-2024-004879 online supplemental file 1

10.1136/spcare-2024-004879 online supplemental file 2

## Data Availability

No data are available.
