# Supplementary material for: The Surprise Question and clinician-predicted prognosis: systematic review and meta-analysis
Source: BMJ Support Palliat Care. 2024 Jun 26;15(1):e004879. doi: 10.1136/spcare-2024-004879 (PMC11874281; doi:10.1136/spcare-2024-004879)
Supplement: online supplemental file 1 [file spcare-15-1-s001.pdf]

Prognostic accuracy of the Surprise Question: a systematic review and meta-analysis.

Citation

Sam Straw, Ankit Gupta, Klaus Witte. Prognostic accuracy of the Surprise Question: a systematic review and meta-analysis.. PROSPERO 2022 CRD42022298236 Available from:
https://www.crd.york.ac.uk/prospero/display_record.php?ID=CRD42022298236

Review question

How accurate is the Surprise Question as a prognostic tool?

Searches

PubMed, OVID (MEDLINE, Embase, AMED, HMIC, Emcare), Web of Science and the Cochrane Database of Systemic Reviews. All will be searched from the start of the database to the present date. The reference list of included in studies and relevant review articles will also be manually evaluated.

Search strategy

https://www.crd.york.ac.uk/PROSPEROFILES/298236_STRATEGY_20211216.pdf

Types of study to be included

Inclusion: studies which report original data pertaining to the accuracy of the Surprise Question, i.e. data provided on response to the question and subsequent mortality which allows calculation of sensitivity, specificity, positive and negative predictive values. Exclusion: studies which do not report original data, e.g. systematic reviews, meta-analyses, editorials, opinion articles, etc. although the references of review articles will be evaluated to identify additional papers.

Condition or domain being studied

The Surprise Question has been proposed as a tool to identify those at risk of dying within a given time frame (typically 1 year). Although originally intended for chronic illness, we will place no limitations on population studied, whether acute or chronic disease, time frame used or who the response was obtained from.

Participants/population

Participants will be healthcare professionals providing responses to the Surprise Question, we will place no limitations on profession, specialist/non-specialists or level of experience.

Intervention(s), exposure(s)

The exposure is a negative ('no' or 'would not be surprised') response to the Surprise Question: “would you be surprised if this patient/person died within the next [time frame]?”

Comparator(s)/control

Not applicable.

Context

Studies which report responses to the Surprise question and survival.

Main outcome(s)

The reported sensitivity, specificity, positive and negative predictive values of the Surprise Question over a given time frame - typically 1 year.

Measures of effect

Sensitivity, specificity, positive and negative predicted values assessed at the time frame specified by the study.

Additional outcome(s)

Not applicable.

Data extraction (selection and coding)

Studies identified from the database searches will be screened by two reviewers independently. The first selection will be based on the abstract/title use of the Surprise Question (or Gold Standards Framework which includes the Surprise Question as a component). Studies identified will undergo full text review. Studies will be included in the review if they report responses to the surprise question and survival data allowing for the specificity, sensitivity, positive and negative predictive values of the Surprise Question to be calculated for the given time frame. Any discrepancies will be resolved by discussion between reviewers, and if unresolved a third reviewer will be consulted. Extraction: which healthcare professionals provided a response to the surprise question, inpatient or outpatient setting, acute or chronic disease, medical speciality, timeframe used. Information on response to the surprise question, proportion who received a surprised/not surprised (or yes/no) response, who subsequently died/survived. Any discrepancies will be resolved by a meeting of the two reviewers, with a third reviewer being included if the discrepancy is unresolved.

Risk of bias (quality) assessment

Studies will be observational in design and bias will be assessed using the Newcastle-Ottawa Scale. No article will be excluded based on bias or quality alone. Each paper will be assessed by two individual raters. Any discrepancies will be resolved by a meeting of two reviewers, with a third reviewer being included if the discrepancy is unresolved.

Strategy for data synthesis [1 change]

Studies will be included in the review if they report responses to the surprise question ('surprised' or 'not surprised') and outcome data (all-cause mortality) allowing for calculation of the specificity, sensitivity, positive and negative predictive values of the Surprise Question for each study. A narrative synthesis will be represented of the findings from the studies including details on the patient population characteristics, clinician characteristics, time frame, and outcome. The quantitative synthesis will be used for studies which report responses to the Surprise Question and outcomes data, with subgroup analyses. The principle question will be the prognostic accuracy of the Surprise Question. Data of responses and all-cause mortality for included studies will be pooled and two by two tables will be constructed. Simple calculations will be performed to calculate the positive predictive, negative predictive, sensitivity and specificity of the Surprise Question (how accurate is the question in predicting all-cause mortality). In this analysis, a true positive is a patient who received a 'not surprised' response who subsequently dies within the study period, whilst a true negative is a patient who received a 'surprised' response and survives. More detailed statistical analysis is not planned, although sub group analyses will be undertaken as described below. There are no agreed criteria for what an acceptable sensitivity/specificity for the Surprise Question would be regarded as (e.g. >0.7 is acceptable), rather we will report these values and their clinical implications.

Analysis of subgroups or subsets [1 change]

The reported accuracy of the surprise question will be assessed by dividing data by healthcare profession (doctor, nurse, experience, specialist or generalist), patient group (acute or chronic disease), speciality (cancer, cardiovascular, etc), and setting (primary care, hospital outpatient, hospital inpatient). We intend to also perform sub analyses of studies by duration of observation. We anticipate most studies will use an observation period of 1 year (as the Surprise Question was originally designed to do), if sufficient studies are available which do not use this time point we will perform additional analyses for <1 year, 1 year, and >1year.

Contact details for further information

Sam Straw

s.straw@leeds.ac.uk

Organisational affiliation of the review [1 change]

University of Leeds

Review team members and their organisational affiliations

Dr Sam Straw. University of Leeds

Mr Ankit Gupta. University of Leeds

Professor Klaus Witte. University of Leeds

Type and method of review

Meta-analysis, Systematic review

Anticipated or actual start date

01 January 2022

Anticipated completion date

31 December 2022

Funding sources/sponsors

Dr Sam Straw is supported by a British Heart Foundation Clinical Research Fellowship

Grant number(s)

State the funder, grant or award number and the date of award

FS/CRTF/20/24071

Conflicts of interest

Language

English

Country

England

Stage of review

Review Ongoing

Subject index terms status

Subject indexing assigned by CRD

Subject index terms

Humans; Palliative Care; Prognosis

Date of registration in PROSPERO

06 January 2022

Date of first submission

16 December 2021

Stage of review at time of this submission

**Stage**	**Started**	**Completed**
Preliminary searches	Yes	Yes
Piloting of the study selection process	Yes	No
Formal screening of search results against eligibility criteria	No	No
Data extraction	No	No
Risk of bias (quality) assessment	No	No
Data analysis	No	No

The record owner confirms that the information they have supplied for this submission is accurate and complete and they understand that deliberate provision of inaccurate information or omission of data may be construed as scientific misconduct.

The record owner confirms that they will update the status of the review when it is completed and will add publication details in due course.

Versions

06 January 2022
