# Supplementary material for: The Surprise Question and clinician-predicted prognosis: systematic review and meta-analysis
Source: BMJ Support Palliat Care. 2024 Jun 26;15(1):e004879. doi: 10.1136/spcare-2024-004879 (PMC11874281; doi:10.1136/spcare-2024-004879)
Supplement: online supplemental file 2 [file spcare-15-1-s002.pdf]

Table 2: Characteristics of studies included in systematic review.

First author	Year	Country	Time	Setting	Speciality	Respondent(s)	Patients	Patient age (years)	Male patients (%)
**Mahes A.**	2023	USA	1-year	Hospital outpatient	Unselected	Physicians	301	74.7 ± 8.2	67.1
**Lin C.A.**	2023	Brazil	1-year	Hospital outpatient	General medicine	Physicians	840	60.9 ± 14.9	31.9
**Um Y.W.**	2023	South Korea	30-days	Emergency department	Unselected	Physicians	300	70.5 (59.3-81.8)	54
**Dogbey D.M.**	2022	South Africa	6-months	Inpatient	Oncology	Physicians	43	58 (45-68)	35
**Kim S.H.**	2022	South Korea	7-days<br>21-days<br>42-days	Inpatient	Oncology	Physicians	130	66.0 ± 12.2	50.8
**Maes H.**	2022	Belgium	1-year	Hospital inpatient	Unselected<br>Cardiology	Physicians	381	Not reported	81.4
**Gaffney L.**	2022	Ireland	1-year	Emergency department	Unselected	Physicians	191	79 (74-83)	45
**Ikari T.**	2021	Japan, Korea, Taiwan	1-day	Inpatient	Oncology	Physicians	1411	72.6 ± 12.2	50.7
**Ikari T.**	2021	Japan, Korea, Taiwan	3-days	Inpatient	Oncology	Physicians	1411	72.6 ± 12.2	50.7
**Moor C.C.**	2021	Netherlands	1-year	Outpatient	Respiratory	Physicians<br>Specialist nurses	140	74.0 ± 6.5	87.1
**Gonzalez-Jaramillo V.**	2021	Colombia	1-year	Outpatient	Cardiology	Physicians	174	70 (58-77)	55.2
**Tripp D.**	2021	USA	30-days<br>1-year	Inpatient	Respiratory	Physicians<br>Advanced practice providers	428	Not reported	49.1
**Ermers D.J.M.**	2021	Netherlands	1-year	Hospital outpatient	Oncology	Physicians<br>Trainee physicians	379	59.4 ± 15	55.7
**Yarnell C.**	2021	Canada	1-year	Hospital inpatient	General medicine	Physicians<br>Trainee physicians	417	75 (60-85)	52.0

**Ros M.M.**	2021	Netherlands	2-days<br>10-days<br>1-year	Intensive care unit	Unselected	Physicians	3140	63.5 ± 16.6	57.1
**Flierman I.**	2020	Netherlands	1-year	Inpatient	Unselected	Nurses	234	81.2 ± 6.6	48.4
**Van Wijmen M.P.S.**	2020	Netherlands	1-year	Primary care	Unselected	Physicians	57	Not reported	28.4
**Ramer S.J.**	2020	USA	2-years	Hospital outpatient	Nephrology	Physicians<br>Advanced practitioners	377	72 (66-78)	49
**Lai C.-F.**	2020	Taiwan	1-year	Hospital outpatient	Nephrology	Nurses	401	56.2 ± 14	49.9
**Rauh L.A.**	2020	USA	1-year	Hospital outpatient	Oncology	Physician<br>Nurse<br>Advanced practice providers	358	Not reported	Not reported
**Yen Y.-F.**	2020	Taiwan	1-year	Hospital inpatient	Unselected	Nurses	21098	62.8 ± 19.0	53.2
**Ouchi K.**	2019	USA	30-days	Emergency department	Unselected	Physicians	10737	75.9 ± 8.8	48.5
**Verhoef M.J.**	2019	Netherlands	1-year	Emergency department	Oncology	Physicians	245	62 (45-79)	48
**Aaronson E.L.**	2019	USA	1-year	Emergency department	Cardiology	Physicians	193	74.5 ± 12.6	
**Schmidt R.J.**	2019	USA	1-year	Hospital outpatient	Nephrology	Physicians<br>Trainee physicians<br>Nurse practitioners	749	69.3 ± 14.6	50.9
**Lakin J.R.**	2019	USA	2-years	Primary care	Primary care	Physicians<br>Nurses	2611	Not reported	40.9
**Veldhoven C.M.M.**	2019	Netherlands	1-year	Primary care	Unselected	Physicians	292	84 ± 5.5	40.1
**Haydar S.A.**	2019	USA	30-days	Emergency department	Unselected	Physicians	6122	66 (51-79)	51.7
**Straw S.**	2019	UK	1-year	Hospital inpatient	Cardiology	Physicians<br>Trainee-physicians<br>Nurses<br>Nurse practitioners	129	71 ± 14	64
**Rice J.**	2018	Canada	3-months	Nursing home	Unselected	Physicians	301	85.9 ± 9.0	67.8

			6-months			Nurses<br>Support workers			
**Burke K.**	2018	UK	90-days<br>1-year	Hospice	Paediatric	Physicians<br>Nurses<br>Administrators	327	7.6 ± 5.3	56.6
**Ouchi K.**	2018	USA	1-year	Emergency department	Unselected	Physicians	207	75 ± 7.5	51.2
**Liyanage T.**	2018	Australia	1-year	Nursing home	Unselected	Nurses	187	82.4 ± 9.1	44.4
**Mitchell G.K.**	2018	Australia	1-year	Primary care	Unselected	Physicians	4365	Not reported	Not reported
**Ebke M.**	2018	Germany	1-year	Hospital outpatient	Neurorehabilitation	Physicians	236	63 ± 14	57.7
**Mudge A.M.**	2018	Australia	1-year	Hospital inpatient	Unselected	Physicians<br>Nurses	100	60.2 ± 18.9	53.8
**Gulini J.E.H.M.B.**	2018	Brazil	1-day	Intensive care unit	Unselected	Physicians	170	57 ± 15.6	51.2
**Salat H.**	2017	USA	1.9-years	Hospital outpatient	Nephrology	Physician	488	71 (65-77)	49
**Malhotra R.**	2017	USA	6-months<br>1-year	Hospital outpatient	Nephrology	Physician	215	Not reported	55
**Hadique S.**	2017	USA	6-months	Intensive care unit	Unselected	Physician	1043	Not reported	53.7
**Gomez-Batiste X.**	2017	Spain	1-year<br>2-years	Primary care<br>Hospital outpatient<br>Intermediate care centre<br>Nursing home	Unselected	Physician/nurse	1059	Not reported	Not reported
**Javier A.D.**	2017	USA	1-year	Hospital outpatient	Nephrology	Physicians<br>Trainee physicians<br>Nurse practitioners	388	71 (65-77)	49.7
**Amro O.W.**	2016	USA	1-year	Hospital outpatient	Nephrology	Physicians	201	66	52.2
**Lakin J.R.**	2016	USA	1-year	Primary care	Unselected	Physicians	1737	65	39.7

**Carmen J.**	2016	Spain	1-year	Hospital outpatient	Nephrology	Physicians	49	Not reported	Not reported
**Hamano J.**	2015	Japan	7-days<br>30-days	Hospital and community palliative care	Oncology	Physicians	2361	69.1 ± 12.8	57.5
**Feyi K.**	2015	UK	1-year	Hospital outpatient	Nephrology	Physicians<br>Nurses	178	72	63.2
**Moroni M.**	2014	Italy	1-year	Primary care	Primary care/oncology	Physicians	231	70.2 (SE 0.9)	50.6
**O'Callaghan A.**	2014	New Zealand	6-months<br>1-year	Hospital inpatient	Acute medicine	Physicians<br>Nurse practitioners	501	Not reported	Not reported
**Da Silva Gane M.**	2013	UK	1-year	Hospital outpatient	Nephrology	Physician<br>Nurses	3896	Not reported	Not reported
**Pang W.-F.**	2013	China	1-year	Hospital outpatient	Nephrology	Physicians	367	60.2 ± 12.3	55.9
**Haga K.**	2012	UK	1-year	Hospital outpatient	Cardiology	Nurses	138	77 ± 10	66
**Fenning S.**	2012	UK	1-year	Hospital outpatient	Cardiology	Physicians	172	66 ±14	61
**Moss A.H.**	2010	USA	1-year	Hospital outpatient	Oncology	Physicians	826	Not reported	Not reported
**Cohen L.M.**	2010	USA	6-months	Hospital outpatient	Nephrology	Physicians	450	Not reported	56.7
**Moss A.H.**	2008	USA	1-year	Hospital outpatient	Nephrology	Physicians	147	66.4 ± 14.8	55.1
**Barnes S.**	2008	UK	1-year	Primary care	Cardiology	Physicians	231	77	54.1
Age is displayed as mean ± standard deviation or median (interquartile range) where available.									

Table 3: Accuracy of individual studies.

First author	Sample size	Surprise Question results				Diagnostic test results				
		Surprise Question responses	Total	Dead	Alive	Sensitivity (95% CI)	Specificity (95% CI)	Positive predictive value (95% CI)	Negative predictive value (95% CI)	Accuracy (95% CI)
**Rice J.**	301	Not surprised	191	62	134	0.646	0.544	0.316	0.825	0.569
		Surprised	194	34	160	(0.541 – 0.741)	(0.485 – 0.602)	(0.276 – 0.360)	(0.779 – 0.863)	(0.518 – 0.619)
**Ikari T.**	1411	Not surprised	847	232	615	0.820	0.455	0.274	0.910	0.528
		Surprised	564	51	513	(0.770 – 0.863)	(0.425 – 0.4840	(0.259 – 0.289)	(0.886 – 0.929)	(0.502 – 0.554)
**Ikari T.**	1411	Not surprised	1179	636	543	0.944	0.263	0.539	0.836	0.588
		Surprised	232	38	194	(0.923 – 0.960)	(0.232 – 0.297)	(0.528 – 0.551)	(0.786 – 0.877)	(0.562 – 0.614)
**Dogbey D.M.**	43	Not surprised	21	18	3	0.667	0.813	0.857	0.591	0.721
		Surprised	22	9	13	(0.460 – 0.835)	(0.544 – 0.960)	(0.676 – 0.945)	(0.446 – 0.721)	(0.563 – 0.847)
**Moor C.C.**	140	Not surprised	39	19	20	0.679	0.821	0.487	0.911	0.793
		Surprised	101	9	92	(0.477 – 0.841)	(0.738 – 0.887)	(0.372 – 0.604)	(0.856 – 0.946)	(0.716 – 0.857)
**Gonzalez-Jaramillo V.**	174	Not surprised	83	17	66	0.850	0.571	0.205	0.967	0.603
		Surprised	91	3	88	(0.621 – 0.968)	(0.489 – 0.651)	(0.166 – 0.250)	(0.911 – 0.988)	(0.527 – 0.677)
**Tripp D.**	381 (30 days)	Not surprised	19	2	17	0.125	0.953	0.105	0.961	0.919
		Surprised	362	14	348	(0.016 – 0.384)	(0.927 – 0.973)	(0.029 – 0.318)	(0.954 – 0.968)	(0.887 – 0.944)
	365 (365 days)	Not surprised	108	38	70	0.469	0.754	0.352	0.833	0.690
		Surprised	257	43	214	(0.357 – 0.583)	(0.699 – 0.803)	(0.285 – 0.425)	(0.801 – 0.861)	(0.640 – 0.738)
**Flierman I.**	234	Not surprised	135	59	76	0.808	0.528	0.437	0.859	0.615
		Surprised	99	14	85	(0.699 – 0.891)	(0.448 – 0.607)	(0.389 – 0.486)	(0.788 – 0.909)	(0.550 – 0.678)
**Kim S.H.**	130 (7 days)	Not surprised	20	7	13	0.467	0.887	0.350	0.927	0.839
		Surprised	110	8	102	(0.213 – 0.734)	(0.815 – 0.938)	(0.204 – 0.531)	(0.888 – 0.954)	(0.764 – 0.897)
	130 (21 days)	Not surprised	54	37	17	0.529	0.717	0.685	0.566	0.615
		Surprised	76	33	43	(0.406 – 0.649)	(0.586 – 0.826)	(0.579 – 0.775)	(0.493 – 0.636)	(0.526 – 0.699)
	130 (42 days)	Not surprised	100	87	13	0.821	0.458	0.870	0.367	0.754
		Surprised	30	19	11	(0.734 – 0.889)	(0.256 – 0.672)	(0.821 – 0.907)	(0.242 – 0.512)	(0.671 – 0.825)
**Ouchi K.**	10737	Not surprised	3324	685	2639	0.433	0.820	0.206	0.931	0.782
		Surprised	12899	896	12003	(0.409 – 0.458)	(0.813 – 0.826)	(0.196 – 0.217)	(0.928 – 0.933)	(0.776 – 0.788)
**Ermers D.J.M.**	379 (SQ1)	Not surprised	188	103	85	0.873	0.674	0.548	0.922	0.736
		Surprised	191	15	176	(0.799 – 0.927)	(0.614 – 0.731)	(0.501 – 0.594)	(0.879 – 0.950)	(0.689 – 0.780)
	188 (SQ2)	Not surprised	105	42	63	0.408	0.259	0.400	0.265	0.340
		Surprised	83	61	22	(0.312 – 0.509)	(0.170 – 0.365)	(0.339 – 0.465)	(0.196 – 0.348)	(0.273 – 0.413)
**Yarnell C.**	417 (AP – 365 days)	Not surprised	298	143	155	0.894	0.397	0.480	0.857	0.588
		Surprised	119	17	102	(0.835 – 0.937)	(0.337 – 0.460)	(0.452 – 0.508)	(0.789 – 0.906)	(0.539 – 0.635)
		Not surprised	250	132	118	0.825	0.541	0.528	0.832	0.650

	417 (SMR – 365 days)	Surprised	167	28	139	(0.757 – 0.881)	(0.478 – 0.603)	(0.490 – 0.565)	(0.777 – 0.876)	(0.602 – 0.696)
	288 (AP - admission)	Not surprised	53	21	32	0.636	0.875	0.396	0.949	0.847
		Surprised	235	12	223	(0.451 – 0.796)	(0.828 – 0.913)	(0.303 – 0.498)	(0.922 – 0.967)	(0.800 – 0.887)
	288 (SMR – admission)	Not surprised	47	16	31	0.485	0.878	0.340	0.930	0.833
		Surprised	241	17	224	(0.308 – 0.665)	(0.832 – 0.916)	(0.242 – 0.455)	(0.904 – 0.949)	(0.785 – 0.875)
**Van Wijmen M.P.S.**	57	Not surprised	19	18	1	0.692	0.968	0.947	0.790	0.842
		Surprised	38	8	30	(0.482 – 0.857)	(0.833 – 0.999)	(0.720 – 0.992)	(0.677 – 0.870)	(0.721 – 0.925)
**Ramer S.J.**	377	Not surprised	124	45	79	0.672	0.745	0.363	0.913	0.732
		Surprised	253	22	231	(0.546 – 0.782)	(0.693 – 0.793)	(0.307 – 0.423)	(0.881 – 0.937)	(0.684 – 0.776)
**Lai C.-F.**	401	Not surprised	34	18	16	0.529	0.956	0.529	0.956	0.920
		Surprised	367	16	351	(0.351 – 0.702)	(0.930 – 0.975)	(0.388 – 0.667)	(0.939 – 0.969)	(0.889 – 0.945)
**Rauh L.A.**	231 (MD – UPMC)	Not surprised	90	36	54	0.706	0.700	0.400	0.894	0.701
		Surprised	141	15	126	(0.562 – 0.825)	(0.627 – 0.766)	(0.334 – 0.470)	(0.845 – 0.929)	(0.638 – 0.760)
	168 (RN – UPMC)	Not surprised	61	31	30	0.689	0.756	0.508	0.869	0.738
		Surprised	107	14	93	(0.534 – 0.818)	(0.671 – 0.829)	(0.417 – 0.599)	(0.810 – 0.912)	(0.665 – 0.803)
	199 (APP – UPMC)	Not surprised	81	35	46	0.796	0.703	0.432	0.924	0.724
		Surprised	118	9	109	(0.647 – 0.902)	(0.625 – 0.774)	(0.364 – 0.503)	(0.870 – 0.956)	(0.656 – 0.785)
	78 (MD – UVA)	Not surprised	39	19	20	0.864	0.643	0.487	0.923	0.705
		Surprised	39	3	36	(0.651 – 0.971)	(0.504 – 0.766)	(0.392 – 0.584)	(0.805 – 0.972)	(0.591 – 0.803)
	130 (RN – UVA)	Not surprised	82	29	53	0.725	0.411	0.354	0.771	0.508
		Surprised	48	11	37	(0.561 – 0.854)	(0.308 – 0.520)	(0.297 – 0.414)	(0.658 – 0.855)	(0.419 – 0.596)
	22 (APP – UVA)	Not surprised	17	8	9	1.000	0.357	0.471	1.000	0.591
		Surprised	5	0	5	(0.631 – 1.000)	(0.128 – 0.649)	(0.376 – 0.568)		(0.364 – 0.793)
	309 (MD – combined)	Not surprised	129	55	74	0.753	0.686	0.426	0.900	0.702
		Surprised	180	18	162	(0.639 – 0.847)	(0.623 – 0.745)	(0.371 – 0.483)	(0.857 – 0.931)	(0.648 – 0.753)
	298 (RN – combined)	Not surprised	143	60	83	0.706	0.610	0.420	0.839	0.638
		Surprised	155	25	130	(0.597 – 0.800)	(0.541 – 0.676)	(0.368 – 0.473)	(0.786 – 0.880)	(0.580 – 0.692)
	221 (APP – combined)	Not surprised	98	43	55	0.827	0.675	0.439	0.927	0.710
		Surprised	123	9	114	(0.697 – 0.918)	(0.598 – 0.745)	(0.378 – 0.501)	(0.874 – 0.959)	(0.646 – 0.769)
**Verhoef M.-J.**	245	Not surprised	203	172	31	0.891	0.404	0.847	0.500	0.788
		Surprised	42	21	21	(0.839 – 0.931)	(0.270 – 0.549)	(0.815 – 0.875)	(0.373 – 0.628)	(0.731 – 0.837)
**Maes H.**	190 (Acute Geriatric Unit)	Not surprised	66	31	35	0.674	0.757	0.470	0.879	0.737
		Surprised	124	15	109	(0.520 – 0.805)	(0.679 – 0.825)	(0.384 – 0.557)	(0.826 – 0.918)	(0.668 – 0.798)
	189 (Cardiology Unit)	Not surprised	63	23	40	0.622	0.737	0.365	0.889	0.714
		Surprised	126	14	112	(0.448 – 0.775)	(0.659 – 0.805)	(0.285 – 0.453)	(0.840 – 0.924)	(0.644 – 0.778)
**Yen Y.-F.**	21098	Not surprised	2620	799	1821	0.456	0.906	0.305	0.948	0.868
		Surprised	18478	955	17523	(0.432 – 0.479)	(0.901 – 0.910)	(0.291 – 0.319)	(0.946 – 0.950)	(0.864 – 0.873)
	193	Not surprised	103	44	59	0.786	0.569	0.427	0.867	0.632

**Aaronson E.L.**		Surprised	90	12	78	(0.656 – 0.884)	(0.482 – 0.654)	(0.371 – 0.486)	(0.794 – 0.916)	(0.560 – 0.700)
**Schmidt R.J.**	749	Not surprised	173	61	112	0.604	0.827	0.353	0.931	0.797
		Surprised	576	40	536	(0.502 – 0.700)	(0.796 – 0.856)	(0.302 – 0.407)	(0.913 – 0.945)	(0.766 – 0.825)
**Lakin J.R.**	1448 (Nurses)	Not surprised	352	112	240	0.526	0.806	0.318	0.908	0.765
		Surprised	1096	101	995	(0.457 – 0.595)	(0.783 – 0.827)	(0.282 – 0.356)	(0.895 – 0.919)	(0.742 – 0.786)
	1163 (Physicians)	Not surprised	452	143	309	0.794	0.686	0.316	0.948	0.703
		Surprised	711	37	674	(0.728 – 0.851)	(0.656 – 0.715)	(0.291 – 0.343)	(0.932 – 0.961)	(0.675 – 0.729)
**Veldhoven C.M.M.**	292 (SQ1)	Not surprised	161	24	137	0.923	0.485	0.149	0.985	0.524
		Surprised	131	2	129	(0.749 – 0.991)	(0.424 – 0.547)	(0.130 – 0.171)	(0.944 – 0.996)	(0.465 – 0.583)
	161 (SQ2)	Not surprised	22	10	12	0.417	0.912	0.455	0.899	0.830
		Surprised	139	14	125	(0.221 – 0.634)	(0.852 – 0.954)	(0.289 – 0.631)	(0.864 – 0.926)	(0.772 – 0.892)
**Burke K.**	325 (majority vote – 90 days)	Not surprised	36	15	21	0.833	0.932	0.417	0.990	0.926
		Surprised	289	3	286	(0.586 – 0.964)	(0.897 – 0.957)	(0.310 – 0.531)	(0.971 – 0.996)	(0.892 – 0.952)
	306 (majority vote – 365 days)	Not surprised	106	25	81	0.833	0.707	0.236	0.975	0.719
		Surprised	200	5	195	(0.653 – 0.944)	(0.649 – 0.760)	(0.195 – 0.282)	(0.946 – 0.989)	(0.665 – 0.769)
	238 (100% agreement - 90 days)	Not surprised	21	14	7	0.933	0.969	0.667	0.995	0.966
		Surprised	217	1	216	(0.681 – 0.998)	(0.936 – 0.987)	(0.488 – 0.808)	(0.970 – 0.999)	(0.935 – 0.985)
	175 (100% agreement – 365 days)	Not surprised	57	21	36	0.955	0.765	0.368	0.992	0.789
		Surprised	118	1	117	(0.772 – 0.999)	(0.689 – 0.829)	(0.302 – 0.441)	(0.945 – 0.999)	(0.721 – 0.847)
	290 (75 – 100% agreement – 90 days)	Not surprised	26	14	12	0.824	0.956	0.539	0.989	0.948
		Surprised	264	3	261	(0.566 – 0.962)	(0.925 – 0.977)	(0.391 – 0.679)	(0.969 – 0.996)	(0.916 – 0.971)
	236 (75 – 100% agreement – 365 days)	Not surprised	80	24	56	0.923	0.733	0.300	0.987	0.754
		Surprised	156	2	154	(0.749 – 0.991)	(0.668 – 0.792)	(0.250 – 0.355)	(0.953 – 0.997)	(0.694 – 0.808)
	122 (Neurology – 365 days)	Not surprised	28	7	21	0.875	0.816	0.250	0.989	0.820
		Surprised	94	1	93	(0.474 – 0.997)	(0.732 – 0.882)	(0.173 – 0.347)	(0.937 – 0.998)	(0.740 – 0.883)
	27 (Oncology – 365 days)	Not surprised	22	12	10	1.000	0.333	0.546	1.000	0.630
		Surprised	5	0	5	(0.735 – 1.000)	(0.118 – 0.616)	(0.456 – 0.632)		(0.424 – 0.806)
	73 (Congenital – 365 days)	Not surprised	24	4	20	0.667	0.702	0.167	0.959	0.699
		Surprised	49	2	47	(0.223 – 0.957)	(0.577 – 0.807)	(0.093 – 0.282)	(0.882 – 0.987)	(0.580 – 0.801)
**Ouchi K.**	207	Not surprised	102	34	68	0.773	0.583	0.333	0.905	0.623
		Surprised	105	10	95	(0.622 – 0.885)	(0.503 – 0.660)	(0.282 – 0.289)	(0.844 – 0.943)	(0.553 – 0.690)
**Liyanage T.**	187	Not surprised	80	30	50	0.714	0.655	0.375	0.888	0.668
		Surprised	107	12	95	(0.554 – 0.843)	(0.572 – 0.732)	(0.309 – 0.446)	(0.829 – 0.928)	(0.596 – 0.735)
**Mitchell G.K.**	2840 (Intuition)	Not surprised	154	32	122	0.337	0.956	0.208	0.977	0.935
		Surprised	2686	63	2623	(0.243 – 0.441)	(0.947 – 0.963)	(0.159 – 0.268)	(0.973 – 0.980)	(0.925 – 0.944)

	1525 (ST)	Not surprised	179	25	154	0.532	0.896	0.140	0.984	0.885
		Surprised	1346	22	1324	(0.381 – 0.679)	(0.879 – 0.911)	(0.107 – 0.181)	(0.978 – 0.988)	(0.868 – 0.900)
**Ebke M.**	236 (Neurorehabilitation Physicians)	Not surprised	45	17	28	0.500	0.861	0.378	0.911	0.809
		Surprised	191	17	174	(0.324 – 0.676)	(0.806 – 0.906)	(0.273 – 0.496)	(0.879 – 0.935)	(0.753 – 0.857)
	236 (Palliative Care Physicians)	Not surprised	83	23	60	0.677	0.703	0.277	0.928	0.699
		Surprised	153	11	142	(0.495 – 0.826)	(0.635 – 0.765)	(0.219 – 0.344)	(0.887 – 0.955)	(0.636 – 0.757)
**Mudge A.M.**	100	Not surprised	52	16	36	0.889	0.561	0.308	0.958	0.620
		Surprised	48	2	46	(0.653 – 0.986)	(0.447 – 0.670)	(0.249 – 0.374)	(0.860 – 0.989)	(0.518 – 0.715)
**Salat H.**	488	Not surprised	171	56	115	0.644	0.713	0.328	0.902	0.701
		Surprised	317	31	286	(0.534 – 0.744)	(0.666 – 0.757)	(0.281 – 0.378)	(0.874 – 0.925)	(0.658 – 0.741)
**Malhotra R.**	208 (180 days)	Not surprised	203	10	193	0.769	0.010	0.049	0.400	0.058
		Surprised	5	3	2	(0.462 – 0.950)	(0.001 – 0.037)	(0.037 – 0.065)	(0.109 – 0.785)	(0.030 – 0.099)
	189 (365 days)	Not surprised	162	13	149	0.650	0.118	0.080	0.741	0.175
		Surprised	27	7	20	(0.408 – 0.846)	(0.074 – 0.177)	(0.059 – 0.108)	(0.580 – 0.855)	(0.123 – 0.236)
**Hadique S.**	500 (Derivation cohort)	Not surprised	238	148	90	0.822	0.719	0.622	0.878	0.756
		Surprised	262	32	230	(0.758 – 0.875)	(0.666 – 0.767)	(0.577 – 0.665)	(0.839 – 0.908)	(0.716 – 0.793)
	543 (Validation cohort)	Not surprised	204	139	65	0.739	0.817	0.681	0.856	0.790
		Surprised	339	49	290	(0.671 – 0.801)	(0.773 – 0.856)	(0.628 – 0.730)	(0.822 – 0.883)	(0.753 – 0.824)
**Gomez-Batiste X.**	1059 (365 days)	Not surprised	837	268	569	0.937	0.264	0.320	0.919	0.446
		Surprised	222	18	204	(0.902 – 0.962)	(0.233 – 0.297)	(0.309 – 0.332)	(0.877 – 0.947)	(0.416 – 0.476)
	1059 (730 days)	Not surprised	837	373	464	0.914	0.287	0.446	0.842	0.529
		Surprised	222	35	187	(0.883 – 0.940)	(0.253 – 0.324)	(0.432 – 0.460)	(0.792 – 0.882)	(0.498 – 0.559)
**Javier A.D.**	388	Not surprised	137	33	104	0.635	0.691	0.241	0.924	0.683
		Surprised	251	19	232	(0.490 – 0.764)	(0.638 – 0.740)	(0.196 – 0.292)	(0.894 – 0.946)	(0.634 – 0.729)
**Amro O.W.**	201	Not surprised	50	22	28	0.550	0.826	0.440	0.881	0.771
		Surprised	151	18	133	(0.385 – 0.707)	(0.759 – 0.881)	(0.336 – 0.549)	(0.839 – 0.913)	(0.707 – 0.827)
**Lakin J.R.**	1737	Not surprised	114	23	91	0.205	0.944	0.202	0.945	0.896
		Surprised	1623	89	1534	(0.135 – 0.292)	(0.932 – 0.955)	(0.143 – 0.277)	(0.940 – 0.950)	(0.881 – 0.910)
**Hamano J.**	2361 (7 days)	Not surprised	931	282	649	0.847	0.680	0.303	0.964	0.704
		Surprised	1430	51	1379	(0.804 – 0.884)	(0.659 – 0.700)	(0.287 – 0.320)	(0.955 – 0.972)	(0.685 – 0.722)
	2361 (30 days)	Not surprised	1851	1066	785	0.956	0.370	0.576	0.904	0.647
		Surprised	510	49	461	(0.942 – 0.967)	(0.343 – 0.397)	(0.565 – 0.587)	(0.876 – 0.926)	(0.627 – 0.666)
**Feyi K.**	178	Not surprised	58	37	21	0.726	0.835	0.638	0.883	0.803
		Surprised	120	14	106	(0.583 – 0.841)	(0.758 – 0.895)	(0.535 – 0.730)	(0.828 – 0.923)	(0.737 – 0.859)
**Moroni M.**	231	Not surprised	126	87	39	0.837	0.693	0.691	0.838	0.758
		Surprised	105	17	88	(0.751 – 0.902)	(0.605 – 0.772)	(0.629 – 0.746)	(0.768 – 0.890)	(0.697 – 0.811)
**O'Callaghan A.**	501 (180 days)	Not surprised	99	56	43	0.727	0.899	0.566	0.948	0.872
		Surprised	402	21	381	(0.614 – 0.823)	(0.866 – 0.926)	(0.487 – 0.641)	(0.926 – 0.963)	(0.840 – 0.900)

	501 (365 days)	Not surprised	99	67	32	0.626	0.919	0.677	0.901	0.856
		Surprised	402	40	362	(0.527 – 0.718)	(0.887 – 0.944)	(0.593 – 0.751)	(0.876 – 0.920)	(0.823 – 0.886)
**Da Silva Gane M.**	3896	Not surprised	938	281	657	0.496	0.803	0.300	0.904	0.758
		Surprised	2958	285	2673	(0.455 – 0.538)	(0.789 – 0.816)	(0.278 – 0.323)	(0.896 – 0.911)	(0.744 – 0.772)
**Pang W.-F.**	367	Not surprised	109	27	82	0.614	0.746	0.248	0.934	0.730
		Surprised	258	17	241	(0.455 – 0.756)	(0.695 – 0.793)	(0.196 – 0.308)	(0.907 – 0.9540	(0.682 – 0.775)
**Haga K.**	138	Not surprised	120	39	81	0.886	0.138	0.325	0.722	0.377
		Surprised	18	5	13	(0.754 – 0.962)	(0.076 – 0.225)	(0.297 – 0.355)	(0.497 – 0.873)	(0.296 – 0.463)
**Fenning S.**	172	Not surprised	38	6	32	0.353	0.794	0.158	0.918	0.750
		Surprised	134	11	123	(0.142 – 0.617)	(0.721 – 0.854)	(0.084 – 0.277)	(0.886 – 0.941)	(0.678 – 0.813)
**Moss A.H.**	826	Not surprised	131	53	78	0.747	0.897	0.405	0.974	0.884
		Surprised	695	18	677	(0.629 – 0.842)	(0.873 – 0.918)	(0.346 – 0.466)	(0.962 – 0.983)	(0.860 – 0.905)
**Cohen L.M.**	450	Not surprised	71	39	32	0.379	0.908	0.549	0.831	0.787
		Surprised	379	64	315	(0.285 – 0.480)	(0.872 – 0.936)	(0.447 – 0.648)	(0.808 – 0.852)	(0.746 – 0.824)
**Moss A.H.**	147	Not surprised	34	10	24	0.455	0.808	0.294	0.894	0.755
		Surprised	113	12	101	(0.244 – 0.678)	(0.728 – 0.873)	(0.189 – 0.427)	(0.851 – 0.926)	(0.677 – 0.822)
**Ros M.M.**	3140 (ICU stay)	Not surprised	153	98	55	0.363	0.981	0.641	0.942	0.928
		Surprised	2987	172	2815	(0.306 – 0.423)	(0.975 – 0.986)	(0.568 – 0.708)	(0.937 – 0.947)	(0.918 – 0.937)
	3140 (Hospital stay)	Not surprised	252	148	104	0.378	0.962	0.587	0.916	0.889
		Surprised	2888	244	2644	(0.329 – 0.428)	(0.954 – 0.969)	(0.531 – 0.641)	(0.909 – 0.921)	(0.878 – 0.900)
	3140 (365 days)	Not surprised	609	363	246	0.509	0.899	0.596	0.862	0.810
		Surprised	2531	350	2181	(0.471 – 0.546)	(0.886 – 0.910)	(0.562 – 0.629)	(0.852 – 0.871)	(0.796 – 0.824)
**Gulini J.E.H.M.B**	170	Not surprised	89	41	48	0.820	0.600	0.461	0.889	0.665
		Surprised	81	9	72	(0.686 – 0.914)	(0.507 – 0.688)	(0.398 – 0.524)	(0.813 – 0.936)	(0.588 – 0.7350
**Haydar S.A.**	6122	Not surprised	918	107	811	0.682	0.864	0.117	0.990	0.859
		Surprised	5204	50	5154	(0.603 – 0.754)	(0.855 – 0.873)	(0.104 – 0.130)	(0.988 – 0.992)	(0.850 – 0.868)
**Carmen J.**	49	Not surprised	20	7	13	0.778	0.675	0.350	0.931	0.694
		Surprised	29	2	27	(0.400 – 0.972)	(0.509 – 0.814)	(0.234 – 0.487)	(0.796 – 0.979)	(0.546 – 0.818)
**Barnes S.**	231	Not surprised	95	11	84	0.786	0.613	0.116	0.978	0.623
		Surprised	136	3	133	(0.492 – 0.953)	(0.545 – 0.678)	(0.087 – 0.153)	(0.942 – 0.992)	(0.558 – 0.686)
**Straw S.**	114 (Consultants)	Not surprised	64	33	31	0.846	0.587	0.516	0.880	0.675
		Surprised	50	6	44	(0.695 – 0.941)	(0.467 – 0.699)	(0.441 – 0.590)	(0.774 – 0.940)	(0.581 – 0.760)
	128 (Junior Doctors)	Not surprised	65	33	32	0.750	0.619	0.508	0.825	0.664
		Surprised	63	11	52	(0.597 – 0.868)	(0.507 – 0.723)	(0.428 – 0.587)	(0.734 – 0.890)	(0.575 – 0.745)
	89 (Heart Failure Nurses)	Not surprised	60	27	33	0.900	0.441	0.450	0.897	0.596
		Surprised	29	3	26	(0.735 – 0.979)	(0.312 – 0.576)	(0.388 – 0.514)	(0.740 – 0.963)	(0.496 – 0.698)
	123 (Staff Nurses)	Not surprised	50	29	21	0.659	0.734	0.580	0.795	0.707
		Surprised	73	15	58	(0.501 – 0.795)	(0.623 – 0.827)	(0.475 – 0.678)	(0.715 – 0.856)	(0.619 – 0.786)
	119 (2 or more)	Not surprised	62	26	36	0.818	0.577	0.500	0.860	0.659

		Surprised	57	8	49	(0.673 – 0.918)	(0.465 – 0.683)	(0.429 – 0.571)	(0.761 – 0.922)	(0.570 – 0.740)
	129 (3 or more)	Not surprised	54	31	23	0.705	0.729	0.574	0.827	0.721
		Surprised	75	13	62	(0.548 – 0.832)	(0.622 – 0.820)	(0.475 – 0.667)	(0.748 – 0.885)	(0.635 – 0.796)
	129 (4 ‘no’s’)	Not surprised	18	14	4	0.318	0.953	0.778	0.730	0.736
		Surprised	111	30	81	(0.186 – 0.476)	(0.884 – 0.987)	(0.551 – 0.909)	(0.687 – 0.769)	(0.652 – 0.810)
	129 (all ‘no’)	Not surprised	35	23	12	0.523	0.859	0.657	0.777	0.744
		Surprised	94	21	73	(0.367 – 0.675)	(0.766 – 0.925)	(0.514 – 0.777)	(0.716 – 0.827)	(0.660 – 0.817)
	32 (Diabetes)	Not surprised	20	9	11	0.900	0.500	0.450	0.917	0.625
		Surprised	12	1	11	(0.555 – 0.998)	(0.282 – 0.718)	(0.339 – 0.566)	(0.621 – 0.987)	(0.437 – 0.789)
	82 (no diabetes)	Not surprised	44	24	20	0.828	0.623	0.546	0.868	0.695
		Surprised	38	5	33	(0.642 – 0.942)	(0.479 – 0.752)	(0.450 – 0.638)	(0.743 – 0.938)	(0.584 – 0.792)
	35 (IHD)	Not surprised	20	14	6	0.824	0.667	0.700	0.800	0.743
		Surprised	15	3	12	(0.566 – 0.962)	(0.450 – 0.867)	(0.539 – 0.823)	(0.577 – 0.922)	(0.567 – 0.875)
	79 (no IHD)	Not surprised	44	19	25	0.864	0.562	0.432	0.914	0.646
		Surprised	35	3	32	(0.651 – 0.971)	(0.424 – 0.693)	(0.352 – 0.516)	(0.784 – 0.969)	(0.530 – 0.750)
	18 (eGFR < 30)	Not surprised	17	10	7	0.909	0.000	0.588	0.000	0.556
		Surprised	1	1	0	(0.587 – 0.998)	(0.000 – 0.410)	(0.542 – 0.633)		(0.308 – 0.785)
	96 (eGFR > 30)	Not surprised	47	23	24	0.821	0.647	0.489	0.898	0.698
		Surprised	49	5	44	(0.631 – 0.939)	(0.522 – 0.759)	(0.399 – 0.580)	(0.796 – 0.952)	(0.596 – 0.788)
**Mahes A.**	301	Not surprised	136	25	111	0.807	0.589	0.184	0.964	0.611
		Surprised	165	6	159	(0.625 – 0.926)	(0.528 – 0.648)	(0.152 – 0.220)	(0.928 – 0.982)	(0.554 – 0.667)
**Lin C.A.**	840	Not surprised	214	32	182	0.615	0.768	0.149	0.968	0.759
		Surprised	626	20	606	(0.483 – 0.748)	(0.739 – 0.798)	(0.101 – 0.196)	(0.954 – 0.982)	(0.730 – 0.788)
**Um Y.W.**	300	Not surprised	118	25	93	0.833	0.662	0.212	0.973	0.679
		Surprised	182	5	177	(0.653 – 0.944)	(0.603 – 0.718)	(0.176 – 0.253)	(0.942 – 0.988)	(0.623 – 0.731)
**Gaffney L.**	191	Not surprised	56	20	36	0.571	0.769	0.357	0.889	0.733
		Surprised	135	15	120	(0.394 – 0.737)	(0.695 – 0.833)	(0.270 – 0.455)	(0.844 – 0.922)	(0.664 – 0.794)

Sensitivity (the ability of the prompt to successfully identify those patients who were dying); specificity (the ability of the prompt to successfully identify those who were not dying); positive predictive value (the proportion of patients who died when the respondent predicted death); negative predictive value (the proportion of the patients who survived when the respondent predicted survival); accuracy (the proportion of correct predictions amongst all cases).

Table 4: Newcastle-Ottawa scale for included studies.

First author	Representativeness of exposed cohort	Selection of non-exposed cohort	Ascertainment of exposure	Outcome of interest accounted for	Age/gender	Other factors	Ascertainment of outcomes	Length of follow-up	Adequacy of follow-up	Quality of study
**Rice J.**	*	*	*	*	*		*	*	*	Good
**Ikari T.**	*	*	*	*	*	*		*	*	Good
**Dogbey D.M.**	*	*	*	*	*	*	*	*	*	Good
**Moor C.C.**	*	*	*	*	*	*	*	*	*	Good
**Gonzalez-Jaramillo V.**	*	*	*	*	*	*	*	*	*	Good
**Tripp D.**	*	*	*	*	*	*	*	*	*	Good
**Flierman I.**	*	*	*	*			*	*	*	Poor
**Kim S.H.**		*	*	*		*		*	*	Good
**Ouchi K.**	*	*	*	*	*	*	*	*		Good
**Ermers D.J.M.**	*	*	*	*			*	*	*	Poor
**Yarnell C.**	*	*	*	*	*	*	*	*	*	Good
**Van Wijmen M.P.S.**	*	*	*	*	*	*	*	*	*	Good
**Ramer S.J.**	*	*	*	*	*	*	*	*	*	Good
**Lai C.-F.**		*	*	*	*	*			*	Poor
**Rauh L.A.**	*	*	*	*	*	*	*	*	*	Good
**Verhoef M.J.**	*	*	*	*			*	*	*	Poor
**Maes H.**	*	*	*	*	*	*	*	*	*	Good
**Yen Y.-F.**	*	*	*	*	*	*	*		*	Good
**Aaronson E.L.**	*	*	*	*		*	*		*	Good
**Schmidt R.J.**	*	*	*	*	*	*	*	*	*	Good
**Lakin J.R.**	*	*	*	*	*	*	*	*	*	Good

**Veldhoven C.M.M.**	*	*	*	*			*		*	Poor
**Burke K.**	*	*	*	*	*	*	*		*	Good
**Ouchi K.**	*	*	*	*			*	*	*	Poor
**Liyanage T.**	*	*	*	*			*	*	*	Poor
**Mitchell G.K.**	*	*	*	*			*		*	Poor
**Ebke M.**	*	*	*	*	*	*		*	*	Good
**Mudge A.M.**	*	*	*	*			*	*	*	Poor
**Salat H.**	*	*	*	*	*	*	*	*	*	Good
**Malhotra R.**	*	*	*	*				*	*	Poor
**Hadique S.**	*	*	*	*	*	*	*	*	*	Good
**Gomez-Batiste X.**	*	*	*	*	*	*	*	*	*	Good
**Javier A.D.**	*	*	*	*	*	*	*	*	*	Good
**Amro O.W.**	*	*	*	*	*	*	*	*	*	Good
**Lakin J.R.**	*	*	*	*	*	*	*		*	Good
**Hamano J.**		*	*	*	*	*		*	*	Good
**Feyi K.**	*	*	*	*	*	*		*	*	Good
**Moroni M.**	*	*	*	*	*	*	*	*	*	Good
**O'Callaghan A.**	*	*	*	*	*	*	*	*	*	Good
**Da Silva Gane M.**	*	*	*	*	*	*	*	*	*	Good
**Pang W.-F.**	*	*	*	*			*	*	*	Poor
**Haga K.**	*	*	*	*			*	*	*	Poor
**Fenning S.**	*	*	*	*	*	*	*	*		Good
**Moss A.H.**	*	*	*	*	*	*	*	*	*	Good
**Cohen L.M.**	*	*	*	*	*	*		*	*	Good

**Moss A.H.**	*	*	*	*	*	*		*	*	Good
**Ros M.M.**	*	*	*	*	*	*	*	*	*	Good
**Gulini J.E.H.M.B.**	*	*	*	*	*	*	*	*	*	Good
**Haydar S.A.**	*	*	*	*	*	*	*			Poor
**Carmen J.**	*	*	*	*				*	*	Poor
**Barnes S.**	*	*	*	*	*	*	*			Poor
**Straw S.**	*	*	*	*	*	*	*	*	*	Good
**Mahes A.**	*	*	*	*	*	*	*	*	*	Good
**Lin C.A.**	*	*	*	*	*	*	*	*	*	Good
**Um Y.W.**	*	*		*	*	*	*	*	*	Good
**Gaffney L.**	*	*	*	*	*	*	*	*	*	Good

* denotes a study having met a particular quality item, scoring one point. The maximum points a study can score is nine, and the minimum is zero.
